# Labile carbon limits late winter microbial activity near Arctic treeline

**DOI:** 10.1038/s41467-020-17790-5

**Published:** 2020-08-12

**Authors:** Patrick F. Sullivan, Madeline C. Stokes, Cameron K. McMillan, Michael N. Weintraub

**Affiliations:** 1 Environment and Natural Resources Institute, University of Alaska Anchorage, Anchorage, AK 99508 USA; 2 Department of Environmental Sciences, University of Toledo, Toledo, OH 43606 USA

**Keywords:** Carbon cycle, Biogeochemistry

## Abstract

Soil microbial communities remain active during much of the Arctic winter, despite deeply frozen soils. Overwinter microbial activity affects the global carbon (C) budget, nutrient cycling, and vegetation composition. Microbial respiration is highly temperature sensitive in frozen soils, as liquid water and solute availability decrease rapidly with declining temperature. Climate warming and changes in snowpack are leading to warmer Arctic winter soils. Warmer winter soils are thought to yield greater microbial respiration of available C, greater overwinter CO_2_ efflux and greater nutrient availability to plants at thaw. Using field and laboratory observations and experiments, we demonstrate that persistently warm winter soils can lead to labile C starvation and reduced microbial respiration, despite the high C content of most Arctic soils. If winter soils continue to warm, microbial C limitation will reduce expected CO_2_ emissions and alter soil nutrient cycling, if not countered by greater labile C inputs.

## Abstract (web summary)

Soil microbial communities remain active throughout much of the Arctic winter, and Arctic winters are warming dramatically. Here, the authors show that persistently warm winter soils can lead to labile carbon starvation and reduced microbial respiration, despite the high carbon content of most Arctic soils.

## Introduction

The importance of overwinter microbial activity to ecosystem C budgets was first recognized in the sub-alpine forests of Colorado and Wyoming, where relatively deep and often early developing snowpacks maintain soil temperatures of −3 to 0 °C throughout the winter^1^. These lightly frozen to unfrozen soils contain substantial amounts of liquid water, because solutes depress the freezing point, minimizing important physical constraints to microbial activity that are present in deeply frozen soils^2^. In these sub-alpine forests, overwinter soil respiration often exceeds 100 g C/m^2^ and is estimated to account for ~10% of total annual ecosystem respiration^3,4^.

**Fig. 1:**
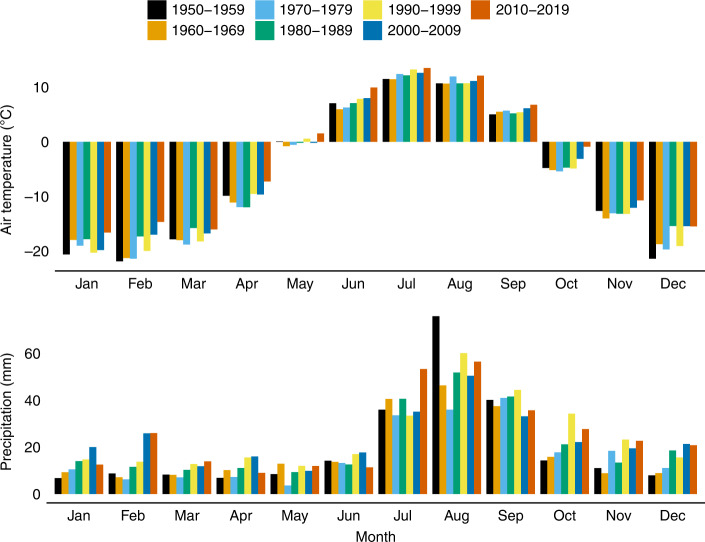
Asymmetric warming and wetting in the western Brooks Range. Long-term air temperature data show more than twice the rate of warming in winter (December–February) than during summer (June–August), whereas long-term precipitation data show increasing trends during the winter months in Kotzebue, AK USA.

Overwinter microbial activity has similarly been observed in the Arctic^5^. However, the combination of very cold air temperatures and shallow snowpacks leads to soils that are deeply frozen throughout most of the snow-covered season. For instance, at Toolik Field Station on the North Slope of Alaska, soil temperature at 10 cm depth was at or below −5 °C for an average of 130 days/year between 1999 and 2017^6^. Estimates of cumulative overwinter soil respiration in the Arctic vary widely, in part because of methodological differences^7^, but they are generally well below the ~100 g C/m^2^ observed in sub-alpine forests^5,7–11^. However, the Arctic is warming at twice the rate of the rest of the globe and the greatest increases in temperature have been observed during the winter months. Long-term air temperature measurements made in Kotzebue, AK (1943–2019) show a rate of winter warming (+0.7 °C/decade, December–February) that is more than twice the rate of summer warming (+0.3 °C/decade, June–August, Fig. 1). Measurements of air temperature in our study area on the Agashashok River (65 km north of Kotzebue) similarly show strong winter warming (Supplementary Fig. 1). Meanwhile, long-term precipitation trends in Kotzebue show increases during the winter months. The combination of strong winter warming and increasing winter precipitation will lead to rapid winter soil warming and may generate biological responses in winter that outpace corresponding changes in the summer.

In comparatively warm sub-alpine forests, sustained microbial activity over the ~6-month snow-covered season can lead to late winter labile C limitation of microbial activity^12^. The development of labile C limitation likely serves as an important negative feedback to warming effects on winter ecosystem-atmosphere C flux—limiting the loss of C that would be expected if soil temperature were the primary constraint on microbial activity. Seasonal development of microbial labile C limitation might also have complex effects on overwinter nutrient cycling and availability to plants early in the growing season, although we are not aware of a study that has tested for these effects.

In deeply frozen Arctic soils, the presence of liquid water is restricted to thin films surrounding soil particles, where the direct and indirect effects of temperature are expected to hold microbial activity at low enough levels to prevent exhaustion of what are generally thought to be large pools of accessible C^13^. However, several lines of evidence point to the potential for labile C limitation of microbial activity in the Arctic. First, a vegetation clipping experiment in Arctic tundra of northern Sweden showed that overwinter microbial respiration is primarily associated with recently fixed plant C, rather than the large pool of bulk soil organic C^14,15^. In this experiment, winter CO_2_ efflux was much lower and less temperature sensitive in clipped than unclipped plots. Second, in sub-Arctic tundra of Alaska, CO_2_ efflux in the Fall was generally greater than in late winter, despite similar soil temperatures^7^. This could reflect a seasonal decline in substrate availability or seasonal differences in liquid soil water availability at a given temperature. Finally, at treeline in northern Sweden, labile C inputs from mountain birch trees were shown to stimulate microbial respiration and decomposition of older soil organic matter, thereby reducing soil C stocks relative to the tundra^16^.

Efforts to model and/or upscale winter C efflux from Arctic ecosystems to the atmosphere recognize the importance of substrate availability for microbial respiration. For instance, in a Pan-Arctic synthesis of winter CO_2_ flux data, leaf area index and gross primary production were important predictors of spatial variation in winter C flux from ecosystems to the atmosphere^11^. Accounting for spatial variation in substrate availability represents an important improvement in our ability to upscale and/or model winter C losses from the Arctic, but it might not address the potential for temporal development of labile C limitation over the course of warmer winters. Along those lines, a biogeochemical model was recently parameterized to account for labile C depletion within the thin water films around particles in deeply frozen soils^17^. The aim was to better reflect the observed rapid decrease in microbial respiration with declining temperature in frozen soils. The revised model allows for temporal development of labile C limitation of microbial respiration at a given soil temperature, but it assumes that a small increase in soil temperature would largely alleviate substrate limitation, as the increase in liquid water would make more labile C available for microbial respiration.

The overarching goal of our study was to improve understanding of controls on overwinter microbial activity in the Arctic, its C cycle feedbacks to climate, and its implications for nutrient availability to plants at snowmelt. Specifically, we used a combination of field observations, field experimentation, and temperature-controlled laboratory incubations to test for the presence of pervasive labile C limitation of microbial respiration near the Arctic treeline. The fieldwork was carried out in three treeline ecotones that differ in soil hydrology (hydric, mesic, and xeric) in the western Brooks Range of Alaska, USA. We also used temperature-controlled laboratory incubations to corroborate and add mechanistic insight to our findings in the field and to explore the potential implications of microbial labile C limitation for nutrient availability to plants. Our results provide conclusive evidence that soil microbes can become limited by labile C availability during late winter, despite the large soil C stocks characteristic of many Arctic ecosystems.

## Results

### Field CO_2_ flux measurements

**Fig. 2:**
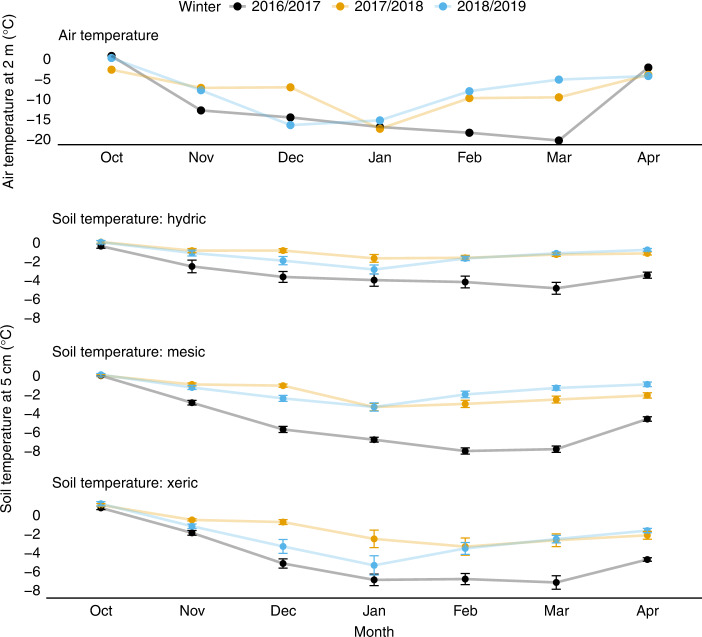
Variation in air and soil temperature across winters. Air temperature was measured at all three sites, but showed negligible variation across sites, as elevation varied by only 35 m. Air temperature data are shown for the Hydric site. Soil temperature is the mean of sensors installed beneath the dripline of 8 control white spruce trees at each site during each winter (bars ± 1.0 S.E.M.). The air temperature data show much colder conditions during November and February–March in the winter of 2016/2017 than during the unusually warm winters of 2017/2018 and 2018/2019. Soil temperature data show persistently warmer soils during the winters of 2017/2018 and 2018/2019, particularly in February–April.

**Fig. 3:**
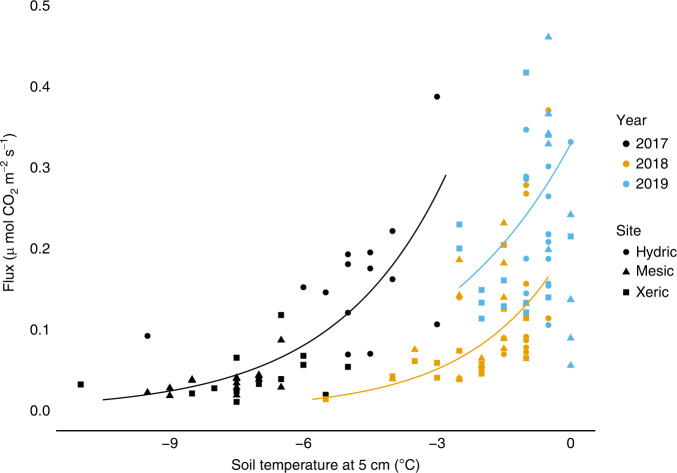
CO_2_ efflux as a function of soil temperature. Measurements were made in three white spruce treeline ecotones in the Agashashok watershed that differ in soil hydrology and soil organic horizon thickness during late March of the relatively cold winter of 2016/2017 and the warm winters of 2017/2018 and 2018/2019. Q_10_ models were fit separately to data from each year. Measurements were made beneath the dripline of both unmanipulated control trees (*n* = 8 per site) and those treated with 1.5 m tall snowfences to increase snow depth (*n* = 8 per site). The results show that CO_2_ efflux at a given temperature was greater near the end of the relatively cold winter of 2016/2017.

Measurements of air temperature, snowpack development, soil temperature, and CO_2_ efflux from soils to the atmosphere were made over three winters at our treeline study sites near the Agashashok River. During the winter of 2016/2017, cold air temperatures in November, coupled with a late-developing snowpack, led to deeply frozen soils throughout the winter (Fig. 2). In contrast, warm air temperatures and early developing snowpacks during the winters of 2017/2018 and 2018/2019 led to warmer soils that were consistently only lightly frozen. Measurements of CO_2_ efflux from soils to the atmosphere made using the diffusion gradient method^18^ in late March of each year showed significantly lower fluxes at a given temperature near the end of the warm winters than in March of 2017, near the end of a relatively cold winter (Fig. 3; Supplementary Fig. 2). Although lower than expected in these relatively warm soils, the CO_2_ fluxes measured in March of 2018 and 2019 were similar in magnitude to measurements made using comparable methods in a sub-alpine forest in Colorado^4^ and far greater than measurements made at lower soil temperatures in the Agashashok study area one-decade earlier^10^. This observational evidence of lower than expected microbial activity near the end of the warm winters could reflect depletion of labile C availability after many months of warm soils conducive to substantial microbial activity.

### Field labile C addition experiment

**Fig. 4:**
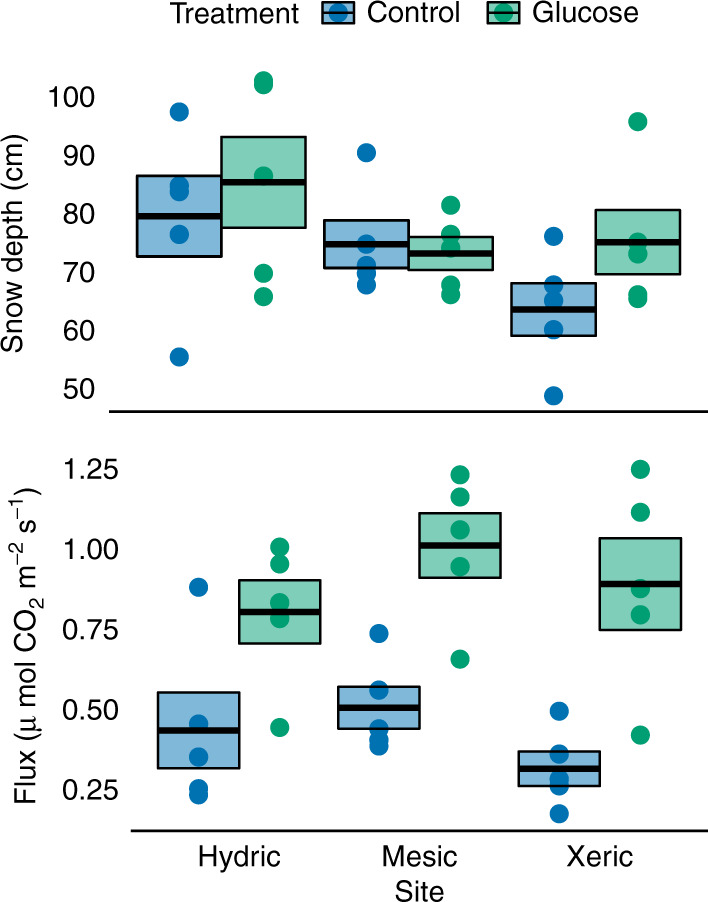
Effects of glucose addition on CO_2_ efflux in the field. Measurements were made 72 h after applying 100 g C/m^2^ in the form of glucose powder to the soil surface of five treatment plots, which were paired with five corresponding control plots, at each of three treeline sites in late March of 2019. Soil surface temperatures were consistent and relatively warm (−2 to −0.5 °C) during the field glucose additions. Pre-treatment snow depths are shown in the upper panel. Analysis of covariance revealed a large effect of glucose addition on CO_2_ efflux (*F*_1,24_ = 52.5, *P* < 0.001, two-sided), which is a proxy for microbial activity. Individual points show plot-level means, while the boxes indicate the mean (center line) and ±1.0 S.E.M.

To test for labile C limitation of microbial activity directly, we conducted experiments in the field and in the laboratory. In the field, we carried out a labile C addition experiment in late March of 2019, during which we added 100 g C/m^2^ of powdered glucose to the soil surface of five treatment plots, which were paired with nearby control plots, at each of our three treeline sites. Soil surface temperatures were consistent and relatively warm (−2 to −0.5 °C) during the field glucose additions. We made pre-treatment measurements of CO_2_ flux from both control and treatment plots and then completely removed the snowpack from both treatment and control plots. After amending the treatment plots with powdered glucose, we immediately returned the snowpack to both control and treatment plots. We tested the optimal timing of treatment response measurements in a boreal forest in Anchorage, Alaska, prior to implementation in the Brooks Range (Supplementary Fig. 3). Testing revealed a large and still increasing CO_2_ flux response to glucose addition after 72 h at our Anchorage test site. CO_2_ fluxes 72 h after glucose application at our Brooks Range treeline sites were doubled from an overall control mean of 0.40 to a glucose treatment mean of 0.89 μmol/m^2^/s (Fig. 4, *F*_1,24_ = 52.5, *P* < 0.001). There was no statistical evidence that the CO_2_ flux response to glucose addition varied across our treeline sites (*F*_2,22_ = 0.25, *P* = 0.780), although there was a trend toward a proportionally greater response at the xeric site, which has less productive understory vegetation and a lower concentration of soil organic C than the mesic and hydric sites (Supplementary Table 1). The results from this field experiment demonstrate that soil microbial respiration was not only temperature limited at the time of sampling.

### Temperature-controlled laboratory incubations

**Fig. 5:**
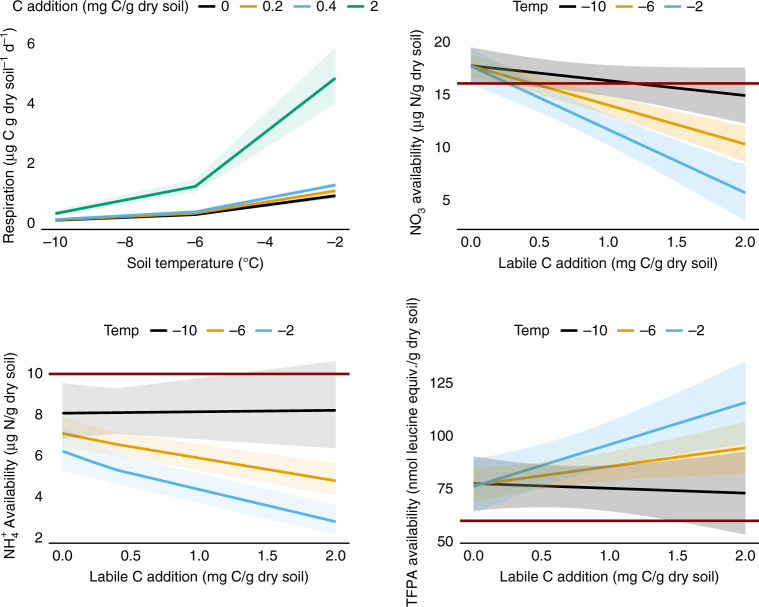
Temperature and labile C addition effects in the laboratory. Effects of temperature and labile C addition on microbial respiration and soil nutrient availability were measured as part of a 90-day laboratory incubation. Cellobiose was added at rates of 0, 0.2, 0.4, and 2 mg C/g dry soil to a homogenized composite of root-free organic soil from our hydric and xeric sites and each was separately incubated at −10, −6, and −2 °C (*n* = 4/temperature*C addition treatment). Respiration was measured at approximately weekly intervals throughout the incubations. Soil NO_3_^−^, NH_4_^+^, and total free primary amine (TFPA) availability were measured before (dark red line) and after the 3-month incubations. The respiration panel shows predictions of a linear mixed effects model, while the nutrient availability panels are effects plots from multiple regression models that include temperature, labile C addition, and their interaction as predictors. Shading indicates 95% confidence intervals for respiration and ±1.0 S.E.M. for nutrient availability.

To gain more mechanistic insight into C limitation to microbial activity at low temperatures than was possible in our field experiment, we conducted temperature-controlled laboratory incubations of soils from our hydric and xeric sites. Soil temperatures were held at −10, −6, −2, 2, and 6 °C and crossed with labile C (cellobiose) additions of 0, 0.2, 0.4 and 2 mg C/g dry soil. Here, we focus on results from incubation temperatures below 0 °C, which best correspond with the range of soil temperatures observed during winter at our field sites. Qualitatively similar results were obtained when all incubation temperatures were included (Supplementary Fig. 4). A linear mixed effects model designed to explain the relationship between measured respiration and the interactive effect of labile C addition and incubation temperature, along with the interaction of incubation temperature with julian date, provided a good fit to the data (predicted vs. observed *r*^2^ = 0.73, observed = predicted*1.01 + 0.01). Over the 3-month incubation, we found strong responses of microbial respiration to labile C addition (Fig. 5, C addition: *t* = 9.5, *P* < 0.001). The magnitude of the microbial respiratory response to labile C addition was small at −10 °C and tended to increase with rising soil temperature (temperature × C addition: *t* = 1.6, *P* = 0.116). Respiration declined during the 3-month incubation (Supplementary Fig. 5, julian date: *t* = −3.2, *P* = 0.002), especially at warmer temperatures (julian date × temperature: *t* = −3.0, *P* = 0.003), indicating rapid utilization of the accessible labile C.

Measurements of soil nutrient availability made at the beginning and end of the incubation suggest that development of labile C limitation will have important implications for overwinter soil nitrogen (N) cycling (Fig. 5; Supplementary Figs. 6–9). Multiple regression models that included incubation temperature and labile C addition amount explained much of the variation in availability of NO_3_^−^ (*r*^2^ = 0.61, *F*_3,44_ = 23.2, *P* < 0.001), and NH_4_^+^ (*r*^2^ = 0.54, *F*_3,44_ = 16.9, *P* < 0.001), while explaining less of the variation in total free primary amine (TFPA) availability (*r*^2^ = 0.23, *F*_3,44_ = 4.4, *P* = 0.008). We also measured orthophosphate-P availability (Supplementary Fig. 10), which was low overall (near detection limits) and showed no evidence of effects of temperature (*t* = −0.7, P = 0.483), nor labile C availability (*t* = 0.7, *P* = 0.504). Among the forms of N, NO_3_^−^ availability increased with warming at lower levels of added C (<~1 mg C/g dry soil, Supplementary Fig. 4), indicating increasing nitrification as a function of temperature. This is consistent with C limitation, because nitrifiers, typically autotrophs that convert NH_4_^+^ to NO_3_^−^, are poor competitors against heterotrophic microbes for NH_4_^+^ and are outcompeted by heterotrophs for NH_4_^+^ when C is available^19–21^. NO_3_^−^ availability declined strongly with labile C addition and the magnitude of decline increased with soil temperature (Temperature*C addition: *t* = −3.5, *P* = 0.001), indicating that warming in the presence of available C promoted net microbial NO_3_^−^ uptake. Similarly, NH_4_^+^ availability declined with labile C addition, but did so only at warmer soil temperatures (Temperature*C addition: *t* = −3.2, *P* = 0.003), indicating that increased C and warmer temperatures stimulated microbial NH_4_^+^ uptake. These results suggest that inorganic N, and especially NO_3_^−^, availability might increase in response to the development of labile C limitation and the associated reduction in microbial N demand. Finally, total free primary amine (TFPA) availability, which is a proxy for organic N availability, increased with labile C addition, but did so only at the warmer soil temperatures (Temperature*C addition: *t* = 2.3, *P* = 0.026), suggesting that changes in organic N availability may be opposite in direction to those for inorganic N in response to labile C limitation. Although our results do not indicate why labile C additions might increase organic N availability, it is possible that added C increased microbial turnover or proteolysis. Collectively, our soil N results suggest that development of labile C limitation will likely have important implications for overwinter N cycling in Arctic soils and may shift the balance of available N forms between NO_3_^−^ and NH_4_^+^ and between inorganic and organic.

## Discussion

Our field observations, field experimentation, and laboratory incubations clearly demonstrate that relatively warm winter soils at our study sites near the Arctic treeline in the western Brooks Range can lead to pervasive labile C limitation of microbial respiration. This finding has important implications for current estimates and projections of Pan-Arctic C budgets with warming, while adding complexity and uncertainty to our understanding of relationships among vegetation, snow and soil nutrient cycling at high latitudes. Recent estimates and projections of Pan-Arctic winter CO_2_ efflux assume that the temperature response of microbial respiration is static over time^11^. For instance, a soil temperature of −3 °C is expected to yield the same CO_2_ flux in March as in November. However, our results, which are consistent with observations in sub-Arctic tundra of Alaska^7^, suggest that a soil temperature of −3 °C in November might yield a higher CO_2_ flux than in March, if soils remain relatively warm during the intervening months. Improved modeling of overwinter C efflux will likely require temperature response models that vary over time with changes in labile C availability. Over longer time periods, labile C limitation of winter microbial respiration could act as an important negative feedback to warming-induced changes in Arctic C budgets. Labile C limitation of microbial respiration may become more common if asymmetric warming leads to an imbalance whereby overwinter increases in microbial activity are not balanced by increases in microbial substrate use efficiency^22^ and/or vegetation productivity and associated labile C production. However, if development of labile C limitation leads to changes in nutrient cycling that yield greater N availability to plants, it is possible that increased vegetation productivity could lead to greater labile C inputs that might prevent further development of labile C limitation.

Vegetation productivity and microbial decomposition of soil organic matter are strongly limited by soil nutrient availability in Arctic tundra^23,24^. The results of our laboratory incubations suggest that development of labile C limitation will have important implications for overwinter soil N cycling. Availability of both NH_4_^+^ and NO_3_^−^ decreased with added labile C, suggesting that labile C limitation might lead to increased inorganic N availability. Meanwhile, amino acid availability tended to increase with added labile C, suggesting that labile C limitation might lead to reduced organic N availability. These changes could have important implications for N availability to plants and microbes, NO_3_^−^ runoff, soil N trace gas emissions associated with nitrification/denitrification, plant productivity, and vegetation community composition. For example, greater overwinter N mineralization could lead to greater soil NO_3_^−^ availability, which could affect early season N losses and N uptake by plants and microbes. Tundra plant species are known to vary in their preference for different forms of N^25^. For instance, sedges of the genus *Carex* are one of the few tundra plants with an apparent preference for NO_3_^−^, while most other tundra plants are thought to favor NH_4_^+^ and/or organic N^26^. Many tundra shrubs form symbioses with mycorrhizal fungi, which facilitate access to more complex forms of organic N^26^. Changes in the magnitude and form of nutrients available to plants may shift the cost/benefit ratio of mycorrhizal associations and alter competitive interactions among plants and between plants and microbes. Growth of treeline white spruce might be more limited by soil P availability than by soil N availability in the Brooks Range^27^. If soil N availability increases, plants and microbes might invest more resources in P acquisition, with implications for soil carbon and nutrient cycling, mycorrhizal associations and plant productivity.

One of the most widespread recent changes in Arctic vegetation is the expansion of tall shrubs into low-statured Arctic tundra^28^. In addition to direct effects of growing season climate warming on shrub growth and reproduction, it is thought that, once established, tall shrubs trap snow, which insulates soils in winter and allows for greater overwinter microbial activity, releasing nutrients that are available to support further shrub growth and expansion^29^. This positive feedback loop among tall shrubs, snow and soil microbes is expected to reinforce the process of tall shrub encroachment. A similar process may operate in treeline environments, where the abrupt change in surface roughness between forest and tundra leads to a reduction in wind speed and both reduced sublimation and increased snow deposition among the trees^10^. Interactions among treeline trees, snow and soil microbes thus have the potential to facilitate treeline advance. Our finding that labile C limitation of microbial respiration can develop over time in lightly frozen Arctic soils and influence both the amount and form of nutrients available to plants adds complexity and uncertainty to the positive feedbacks that are thought to reinforce shrub expansion and treeline advance into Arctic tundra.

While our finding that Arctic soil microbes can become limited by labile C availability represents an important advance in understanding, a number of key uncertainties remain. For instance, the spatial extent of late winter microbial labile C limitation within the Arctic is uncertain. Thus far, all of the evidence comes from sites near the southern limit of Arctic tundra. Field measurements, experimentation and modeling efforts deeper within the Arctic will be important to define the northern limits of overwinter microbial C limitation. Meanwhile, our observations from the northern and southern limits of the boreal forest in Alaska suggest that overwinter microbial labile C limitation might be widespread in the Boreal biome. Studies focused on different vegetation types and more continental areas of the Boreal would similarly help to define the spatial extent. Our understanding of the temporal extent of microbial labile C limitation is also limited at present. It remains unclear when during the winter labile C limitation begins to develop, at what rate it develops and how the timing of development might vary with factors such as vegetation type and associated mycorrhizal^30^ and microbial communities, the size of the labile C pool and soil organic C stocks. These spatial and temporal limits to our understanding constrain our ability to quantify the magnitude of the C cycle implications of overwinter microbial labile C limitation. In addition, our assessment of the implications of microbial C limitation for soil nutrient cycling represents a first look. Further work on this topic in the field and in the laboratory will be important to fully unravel the implications of overwinter microbial labile C limitation for soil nutrient cycling and vegetation change. Thus, we argue that our finding of overwinter microbial labile C limitation highlights an important new avenue for future research in the Arctic and in other ecosystems with seasons of limited photosynthetic labile C production.

## Methods

### Site description

Field measurements and sample collections were made in three diffuse treeline ecotones near the Agashashok River in the Baird Mountains of northwest Alaska. The study area is near the northern and western limits of white spruce (*Picea glauca*) in Alaska. The northernmost tree in the Agashashok watershed is approximately 12 km north of the study area. The hydric treeline (67.47 N, 162.20 W, 155 m asl) has an understory of wet sedge tundra that is dominated by *Carex bigelowii* and mosses of the genera *Sphagnum* and *Hylocomium* with occasional tussocks of *Eriophorum vaginatum*. The mesic treeline (67.48 N, 162.22 W, 135 m asl) is typical tussock tundra, dominated by *Eriophorum vaginatum*, *Betula nana, Rhododendron groenlandicum, Vaccinium uliginosum, Salix pulchra* and mosses of the genera *Hylocomium, Pleurozium*, and *Sphagnum*. The xeric treeline (67.47 N, 162.21 W, 170 m asl) has an understory of dry heath tundra. The most common plant species at the xeric site is *Dryas octopetala*, with abundant lichens and lesser amounts of *Cassiope tetragona* and *Empetrum nigrum*. The area is ~65 km north of Kotzebue, Alaska and is accessed using bush planes in the summer and snowmachines in the winter.

The study sites are part of a snowfence experiment, designed to examine the role of winter snow depth as a driver of treeline tree growth and reproduction. Snowfences (1.5 m tall, 7 m long) were installed 2 m upwind of eight white spruce trees (~5 cm dbh, ~3.5 m tall) at each site in early September of 2016. Each of the three sites is equipped with a meteorological station that records hourly air temperature and snow depth, along with soil temperature and soil moisture at 10 cm depth increments. iButton temperature loggers (DS1921G) are installed at 5 cm depth beneath the dripline of 8 control and 8 snowfence trees at each site (48 trees total). iButtons were programmed to record soil temperature at 4-h intervals during the winter months.

Long-term air temperature data for Kotzebue, Alaska were acquired from the National Center for Environmental Information at the National Oceanic and Atmospheric Administration (https://www.ncdc.noaa.gov/). Long-term precipitation data for Kotzebue were acquired from the Alaska Climate Research Center at the University of Alaska Fairbanks (http://akclimate.org/). Measurements of air temperature at 2 m height have been made since June of 2006 on a riverside terrace in the Agashashok watershed^31^ using a CS215 sensor (Campbell Scientific, Logan, UT, USA) that is housed within a 6-plate radiation shield (R.M. Young, Traverse City, MI, USA). The sensor is scanned every 15 min and hourly averages are logged to a CR1000 datalogger (Campbell Scientific, Logan, UT, USA).

### Field CO_2_ flux measurements

The study sites were visited during the final week of March in 2017, 2018, and 2019, and measurements of CO_2_ efflux were made at the dripline of each study tree using the diffusion gradient method^18^ during periods with light winds. Measurements of atmospheric [CO_2_] at the snow surface and subnivean [CO_2_] at the ground surface were made at each study tree using a hollow stainless-steel probe that was etched with snow depth increments (Snowmetrics, Fort Collins, CO) and plumbed with 3.2 mm I.D. polyethylene tubing. The tubing was fitted to the inlet of a LI-840 NDIR CO_2_ and H_2_O analyzer (LI-COR Environmental, Lincoln, NE), which was equipped with a micro-diaphragm pump (850 ml/min, KNF Neuberger Inc., Trenton, NJ) downstream of the optical bench. The zero of the LI-840 was checked daily before initiating field measurements, while regular measurements of atmospheric [CO_2_] provided an opportunity to check for analyzer drift. Readings of subnivean [CO_2_] consistently stabilized and were recorded within 1 min of probe insertion. In addition to measurements of atmospheric and subnivean [CO_2_] at each study tree, measurements were also regularly made of [CO_2_] at 10 cm intervals throughout the snowpack to test for potential barriers to diffusion that could lead to overestimates of CO_2_ efflux when using the 2-point diffusion method^18^.

After completing the [CO_2_] and snow depth measurements at each tree, three snow pits were excavated in representative areas within the treeline ecotone at each site. Measurements of snow density and temperature were made in continuous 10 cm intervals along the walls of the snow pits using a RIP 1 density cutter (Snowmetrics, Fort Collins, CO) and a 600 g capacity spring scale with 5 g resolution (Pensola, Schindellegi, Switzerland). Corresponding measurements of snow temperature were made using a dial stem thermometer.

Diffusion of CO_2_ from the subnivean to the atmosphere was estimated as follows^32^:

$$J_{\mathrm{c}} = \theta \tau D\frac{{P_0}}{{RT_0}}\left( {\frac{T}{{T_0}}} \right)^{0.81}\frac{{\Delta C}}{z},$$

where *J*_c_ is CO_2_ efflux (µmol/m^2^/s), *θ* is snowpack porosity (unitless), *τ* is snowpack tortuosity (unitless), *D* is the diffusion coefficient for CO_2_ in air (0.1381 × 10^−4^/m^2^/s), *P*_0_/*RT*_0_ is the molecular density of CO_2_ at standard temperature and pressure (44.613 mol/m^3^), *T* is snowpack temperature (K), Δ*C* is the difference in [CO_2_] between the subnivean and the atmosphere (µmol/mol), and *z* is snow depth (m). Snowpack porosity (*θ*) was estimated using mean snowpack density (*ρ*):

$$\theta = 1\frac{P}{{973}},$$

where 973 g/l is the density of ice. Snowpack tortuosity (*τ*) was also estimated as a function of density^33^:

$$\tau = \theta ^{1/3}.$$

### Field labile C addition experiment

During the final week of March 2019, 250 g/m^2^ of glucose powder (Product # G8270, Sigma Aldrich, Saint Louis, MS) was added to the soil surface of five 1.0 m^2^ treatment plots, which were paired with five control plots, in each treeline ecotone. The magnitude of the glucose addition was set to match that of a similar earlier experiment in the sub-alpine forest of Colorado^12^. Prior to snowpack disturbance and glucose application, pre-treatment measurements of CO_2_ efflux were made from each control and treatment plot. The snowpack was then carefully removed from a 2.25 m^2^ area centered on each control and treatment plot. After removing the more consolidated upper portion of the snowpack, the large, faceted depth hoar crystals were swept off the plot using a whisk broom. Glucose power was evenly spread over the central 1.0 m^2^ area of the treatment plots. The snowpack was then returned to the control and treatment plots, first by sweeping the depth hoar back on to the plot and then by returning the blocks of consolidated upper snowpack. The snowpack was lightly packed using snowshoes and any cracks visible at the snow surface were filled with fresh snow. After 72 h, measurements of CO_2_ efflux were made using the aforementioned diffusion method near the center of each control and glucose treatment plot. After completing the [CO_2_] measurements, snow pits were excavated for measurements of snow density and temperature as described above at each control and treatment plot in each treeline ecotone.

### Temperature-controlled laboratory incubations

Organic horizon tundra soils were collected to a depth of 15 cm in summer of 2018 from the hydric and xeric treeline sites. Soils were frozen and shipped to Toledo, OH, USA, where they remained frozen at −20 °C until initiation of the incubation experiment. Soils were thawed and rocks, large roots and woody debris were removed before the soils were homogenized into a single composite with roughly three parts hydric to one part xeric, reflecting the quantity of available material. Soils were then kept in a 4 °C incubator at a near constant soil moisture of ~50% water holding capacity for roughly three weeks.

To determine the effects of labile C availability and temperature on respiration, the composite soil was incubated with different amounts of labile C at a range of temperatures. Adding C as cellobiose (a dimer of glucose) rather than glucose allowed us to measure changes in the activity of the enzyme that cleaves this dimer (beta-glucosidase) and determine whether its production was upregulated during the incubation. Beta-glucosidase activities at the end of the incubation did not vary as a function of C addition or temperature and are not shown. Incubations were initiated by placing 25 g (wet mass) of composite soils into 80 half pint wide mouth canning jars (Jarden Corporation, Muncie, IN, USA) fitted with septa, and maintained at 4 °C. 5 mL of cellobiose solution at 4 °C was added at concentrations of 0, 0.5, 1, or 5 mg C/mL in reagent grade water (*n* = 20/cellobiose concentration). This approach achieved C additions of 0, 0.2, 0.4, and 2 mg C/g dry soil, respectively. Samples of each C addition treatment were loosely covered with jar lids to prevent CO_2_ buildup and immediately placed in lab incubators set at −10, −6, −2, 2, and 6 °C (*n* = 4/temperature*C addition).

A LI-820 infrared gas analyzer (LI-COR Biosciences, Lincoln, NE, USA) outfitted for static injections was used to measure respiration every 3–4 days in the first month, once a week in the second month, and every other week during the third and final month of the incubation. While measuring respiration, the sample masses were taken and water losses were found to be negligible. To measure respiration, samples were uncovered and vented using a desk fan to clear the headspace and then sealed. To allow enough time for detectable concentrations of CO_2_ to accumulate in jar headspaces, the samples at below-freezing temperatures had to be incubated for 3–4 days, while those at above-freezing temperatures only required 3 h. While measuring respiration from below-freezing incubations, jars were kept on ice. A minimum of two 2 mL headspace samples per jar were analyzed (a third was analyzed if the first two were inconsistent). CO_2_ concentrations were estimated using a three-point calibration curve ranging from 0 to 5000 ppm CO_2_, and respiration is reported as μg C g dry/soil/day.

At the end of the 3-month incubation, soils were extracted for carbon and nutrient analysis. 5 g of soil was combined with 25 mL of 0.5 M K_2_SO_4_ in a 50 mL tube and placed on an orbital shaker for 1 h. The sample was then filtered using a Whatman #1 paper filter (2 µm pore size) and a vacuum filtration system^34^. Total reducing sugar (TRS) concentrations (Supplementary Figure 11) in the unfumigated extracts were determined in four analytical replicates per sample using a colorimetric microplate assay with glucose as the standard and expressed in terms of glucose equivalents^35,36^. The detection limit of the TRS assay was 5 μg glucose equiv./mL. NH_4_^+^-N and NO_3_^−^-N were measured on three analytical replicates per sample with colorimetric microplate assays^37,38^. Detection limits in both assays were 0.1 μg N/mL. Total free primary amines (TFPA) were quantified using a fluorometric microplate assay with leucine as the standard and expressed in terms of leucine equivalents, with a detection limit of 0.5 µmol leucine equiv./mL^39,40^, .

Microbial biomass (Supplementary Figs. 12 and 13) was determined using a modification of the chloroform fumigation extraction method^41,42^, in which 2 mL of chloroform was added to 5 g of soil in a 250 mL Erlenmeyer flask, and then immediately stoppered. After 24 h the stoppers were removed, and the chloroform vented in a fume hood for 30 min Samples were then extracted as described above.

Dissolved organic C (DOC) and total dissolved nitrogen (TDN) concentrations were measured using a Shimadzu total organic C (TOC-V_CPN_) analyzer with a total nitrogen (TN) module (Shimadzu Scientific Instruments Inc., Columbia, MD, USA), which has a detection limit of 0.5 ppm organic C, and 0.1 ppm N. Extractable orthophosphate-P was measured on three analytical replicates per sample with a colorimetric microplate assay that has a detection limit of 0.01 µg PO_4_-P/mL^43^, . All microplate assays were read on Bio-Tek Synergy HT microplate reader (Bio-Tek Inc., Winooski, VT, USA). TOC, TN, and P concentrations in unfumigated extracts were subtracted from those in fumigated extracts to estimate extractable microbial biomass C, N, and P. Extractable microbial biomass C, N, and P were not corrected for extraction efficiency, which is unknown in these soils, and are expressed as μg (C, N, or P)/g dry soil.

### Statistical analyses

Data processing, statistical analyses, and data visualization were performed using R 4.0.2^44^. All statistical tests are two-sided. Q_10_ temperature response models were fit to tree-level soil temperature and CO_2_ efflux estimates across sites, separately for each year, using the nls function in R:

$${\mathrm{CO}}_2\,{\mathrm{flux}} = A \times B^{\left( {T + 5} \right)/10},$$

where *A* is the estimated CO_2_ flux at −5 °C, *B* is the Q_10_ (the factor by which CO_2_ flux increases for a 10 °C increase in soil temperature), and *T* is the measured soil temperature (°C). Model predictions for each year at a common soil temperature of −3 °C were generated using the predictNLS function in the propagate package^45^. Monte Carlo simulations were performed to estimate the confidence intervals around the predictions at alpha = 0.05.

The effect of experimental glucose addition to field plots was examined using an analysis of covariance, with the pre-treatment CO_2_ flux, site (hydric, mesic, xeric), and treatment (control, glucose) as the main effects:

$${\mathrm{ln}}({\mathrm{CO}}_2{\mathrm{flux}}_{{\mathrm{post}}}) = \alpha + \beta _1({\mathrm{ln}}({\mathrm{CO}}_2{\mathrm{flux}}_{{\mathrm{pre}}})) + \beta _2\left( {{\mathrm{Site}}_{{\mathrm{mesic}}}} \right) \\ + \beta _3\left( {{\mathrm{Site}}_{{\mathrm{xeric}}}} \right) + \beta _4({\mathrm{Treatment}}) + {\it{\epsilon }},$$

where *α* is the intercept and *β*_1–4_ are the coefficients for each term in the model and ${\it{\epsilon }}$ is the Gaussian error term. The CO_2_ flux measurements were natural log-transformed prior to analysis to improve conformity with model assumptions. Interactions between site and treatment (*F*_2,22_ = 0.25, *P* = 0.780) and between pre-treatment CO_2_ flux and treatment (*F*_1,23_ = 0.30, *P* = 0.591) were tested and excluded from the final model.

Respiration data from the temperature-controlled laboratory incubations were analyzed for all incubation temperatures and for just those below 0 °C using a linear mixed effects model in the nlme package^46^. The respiration data were natural log-transformed to linearize the relationship between temperature and respiration. A random intercept for sample was included in the model. For 13 of the 1072 observations, respiration was below detection limits. These observations were assigned a flux of 0.01 μg C g dry/soil/day, which was consistent with the lowest detectable flux. The model included the interaction of incubation temperature and C addition, the interaction of julian date and temperature and the associated main effects:

$${\mathrm{ln}}\left( {{\mathrm{Respiration}}} \right)_{ij} = \, \alpha + \beta _1\left( {{\mathrm{sample}}_i} \right) + \beta _2\left( {{\mathrm{temperature}}_{ij}} \right) + \beta _3\left( {{\mathrm{C}}\,{\mathrm{addition}}_{ij}} \right)\\ \, + \beta _4\left( {{\mathrm{date}}_{ij}} \right) + \beta _5({\mathrm{temperature}}_{ij} \times {\mathrm{C}}\,{\mathrm{addition}}_{ij})\\ \, + \beta_6({\mathrm{temperature}}_{ij} \times {\mathrm{date}}_{ij}) + {\it{\epsilon }}_{ij},$$

where LN(Respiration)_*ij*_ is natural log-transformed respiration during the *j*th measurement of the *i*th sample, *α* is the intercept, *β*_1–6_ are the fixed coefficients and ${\it{\epsilon }}$_*ij*_ is the Gaussian error term. The three-way interaction of temperature, julian date, and C addition was tested, but excluded, as it was not significant when the model was fitted to all of the incubation temperatures, nor when the analysis was restricted to just temperatures below 0 °C. The predict function in R was used to make predictions of respiration at each level of labile C addition, for each temperature. The predictions and associated standard errors were transformed back to their original scale for plotting.

Measurements of soil nutrient availability from the end of the incubations were analyzed using multiple regression models that included temperature, the amount of labile C addition and their interaction:

$${\mathrm{Nutrient}}\;{\mathrm{availability}} = \alpha + \beta _1({\mathrm{temperature}}) + \beta _2({\mathrm{C}}\;{\mathrm{addition}})\\ +\, \beta _3({\mathrm{temperature}} \times {\mathrm{C}}\;{\mathrm{addition}}) + {\it{\epsilon }},$$

One of the observations of NH_4_^+^-N availability was below detection limits. This observation was assigned a value of 0.5 μg N/g dry soil, which was consistent with the lowest measured NH_4_^+^-N availability. The NH_4_^+^-N data were then natural log-transformed to improve conformity with model assumptions. Interaction plots were generated using the interact_plot function in the interactions package^47^. All graphics were produced using the ggplot2 package^48^.

### Reporting summary

Further information on research design is available in the Nature Research Reporting Summary linked to this article.

## Supplementary information

Supplementary Information

Peer Review File

Reporting Summary

## Data Availability

Data presented in this article have been archived in the Arctic Data Center of the National Science Foundation, USA: 10.18739/A2V40K067.

## Code availability

R scripts written to analyze the data and produce the graphics have been archived along with each of the datasets in the Arctic Data Center of the National Science Foundation, USA: 10.18739/A2V40K067.
