# Supplementary material for: Labile carbon limits late winter microbial activity near Arctic treeline
Source: Nat Commun. 2020 Aug 12;11:4024. doi: 10.1038/s41467-020-17790-5 (PMC7423931; doi:10.1038/s41467-020-17790-5)
Supplement: Supplementary file 1 — Supplementary Information [file 41467_2020_17790_MOESM1_ESM.pdf]

Supplementary Information

Labile carbon limits late winter microbial activity near Arctic treeline

Patrick F. Sullivan et al.

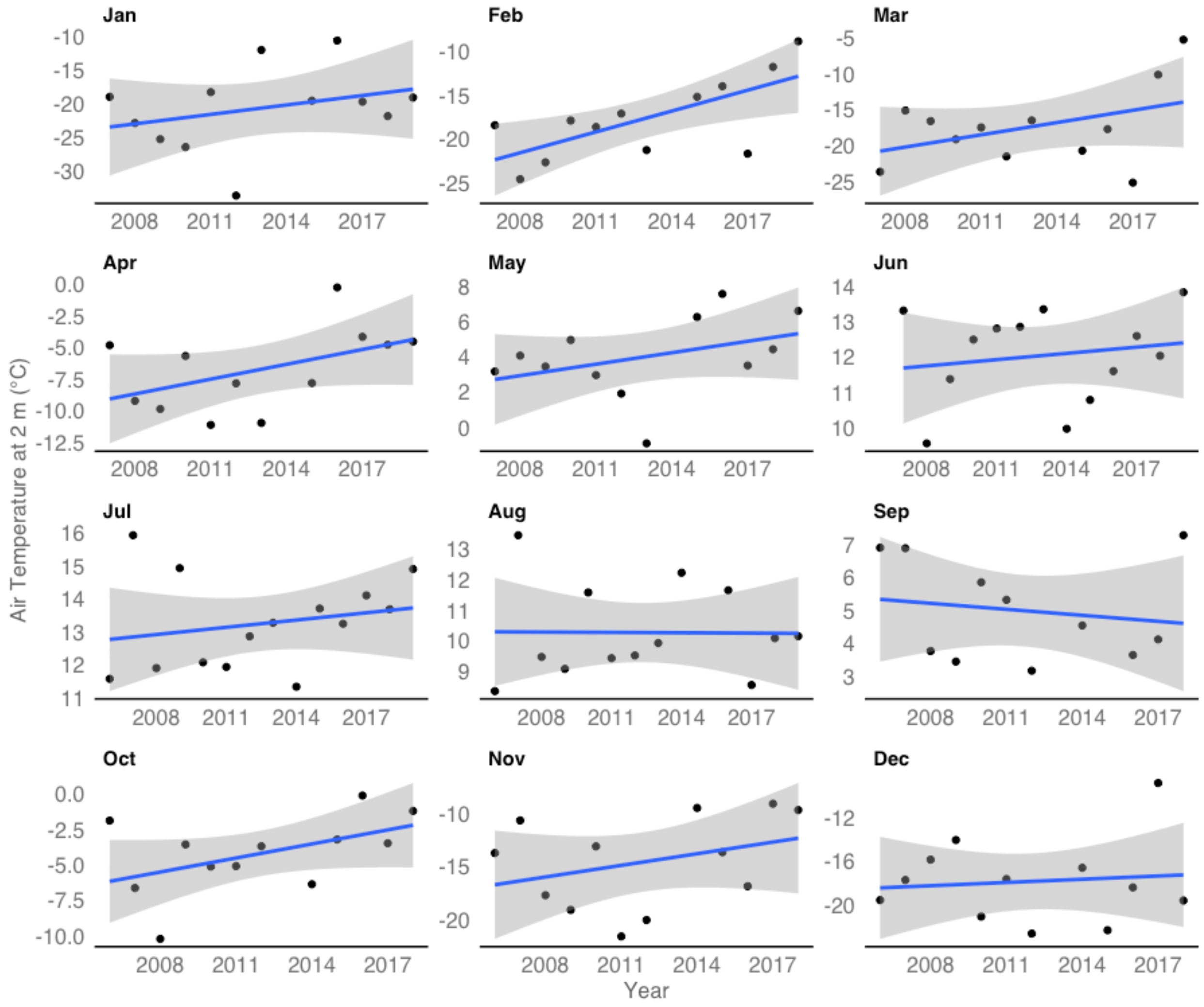

Supplementary Figure 1. Recent asymmetric warming in the Agashashok River study area. The February air temperature trend is statistically significant, despite the relatively short record (n=12 years, r^2=0.48, P=0.012). Grey shading indicates ± 1.0 S.E.M.

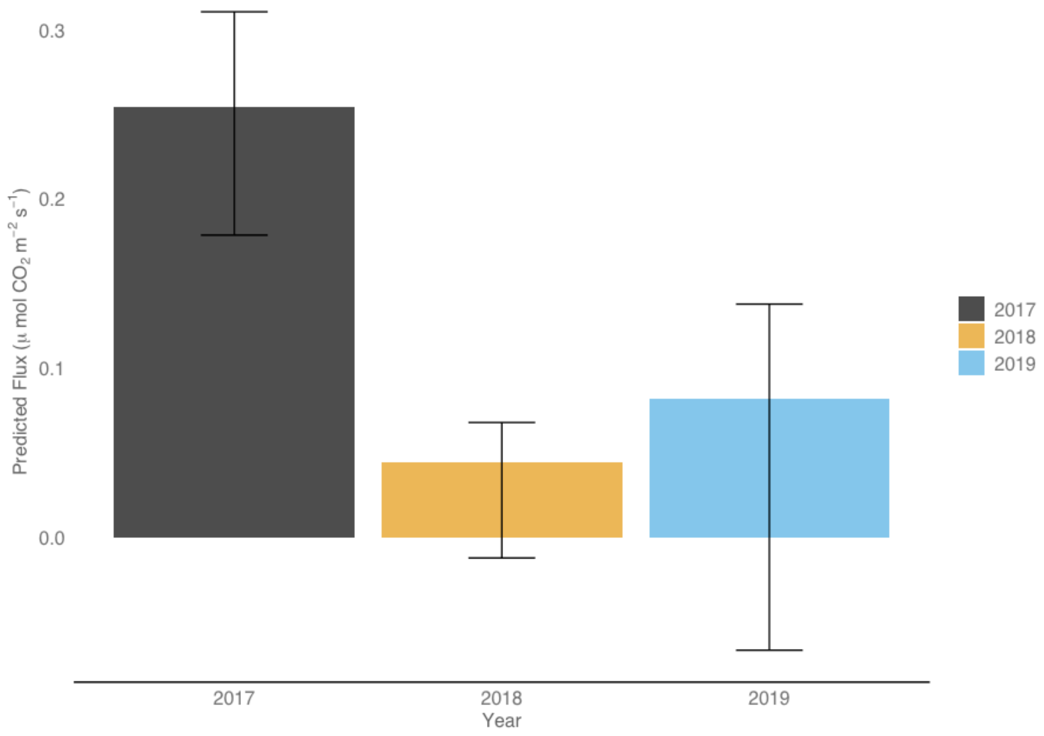

Supplementary Figure 2. Predicted late winter CO_2 flux at a soil temperature of -3°C. Predictions were based on separate Q_{10} model fits to the data from each year. The filled bars represent the mean predictions for each year, while the error bars represent 95% confidence intervals estimated by Monte Carlo simulation (n=1,000,000 simulations). The non-overlapping confidence intervals indicate that fluxes were significantly greater near the end of the relative cold 2016/2017 winter than near the ends of the relatively warm winters of 2017/2018 and 2018/2019.

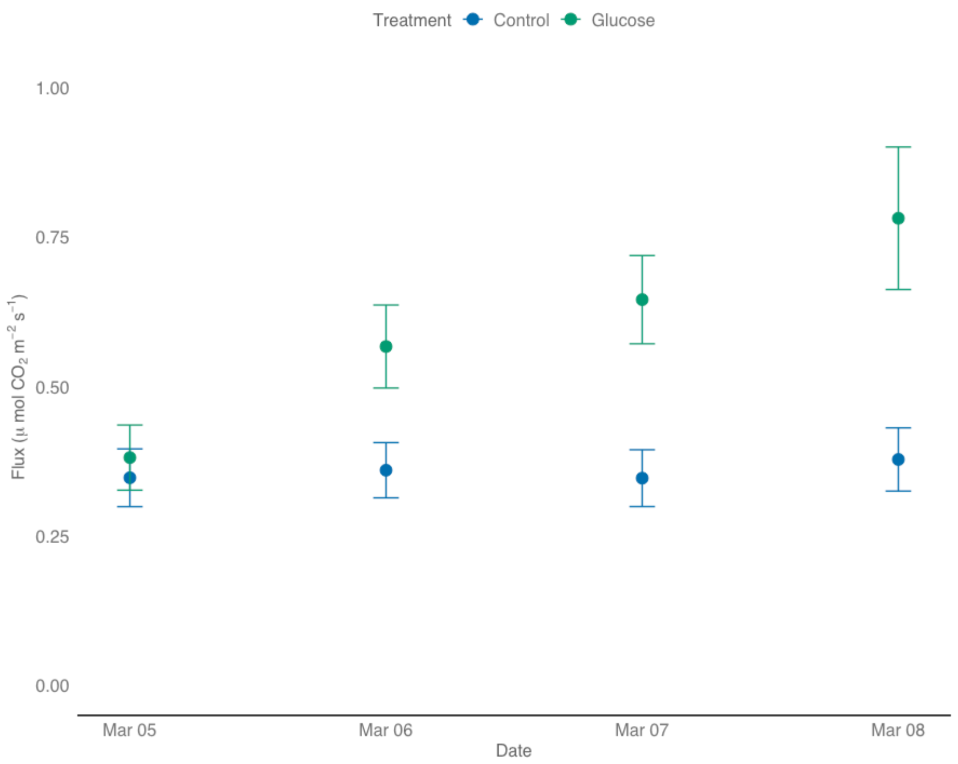

Supplementary Figure 3. Effects of glucose addition on CO_2 flux in a boreal forest. The response of CO_2 flux to experimental glucose addition was monitored over time in a mixed white spruce, black spruce, paper birch forest in Anchorage, Alaska. Pre-treatment CO_2 flux measurements were made on March 5, 2019 and were immediately followed by snowpack removal from paired control (n=5) and treatment plots (n=5). Powdered glucose was added to the treatment plots (100 g C/m^2) and the snowpack was returned to both control and treatment plots. Measurements of CO_2 flux were made using the diffusion method at 24, 48 and 72 hours after glucose addition. Snow pits were excavated for measurements of snow density and temperature after the final round of measurements and applied to the [CO_2] measurements made at 24 and 48 hours. Daytime maximum and nighttime minimum air temperatures were consistent over the 72-hour period. Soil surface temperature was -2°C at the beginning and end of the experiment. Bars are ± 1.0 S.E.M.

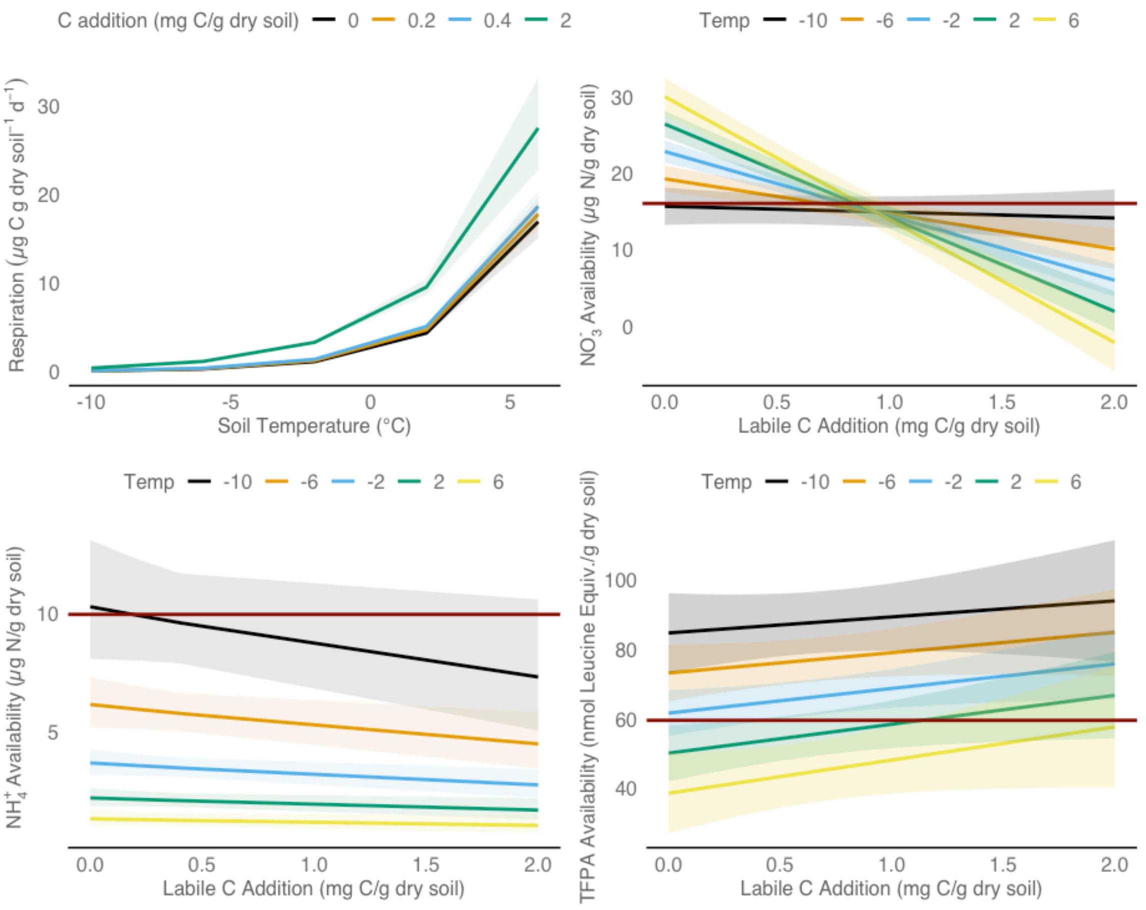

Supplementary Figure 4. Temperature and labile C addition effects in the laboratory. Effects of temperature and labile C addition on microbial respiration and soil nutrient availability were measured as part of a ~90-day laboratory incubation. Cellobiose was added at rates of 0, 0.2, 0.4, and 2 mg C per g dry soil to a homogenized composite of root-free organic soil from our hydric and xeric sites and each was separately incubated at -10, -6, -2, 2, and 6 °C (n=4 samples/temperature*C addition treatment). The respiration data were analyzed using a linear mixed effects model. Soil nutrient availability was measured before (dark red line) and at the end of the three-month incubations. The respiration panel shows predictions of a linear mixed effects model, while the nutrient availability panels are effects plots from multiple regression models that include temperature, labile C addition and their interaction as predictors. Shading indicates 95% confidence intervals for respiration and ± 1.0 S.E.M. for nutrient availability.

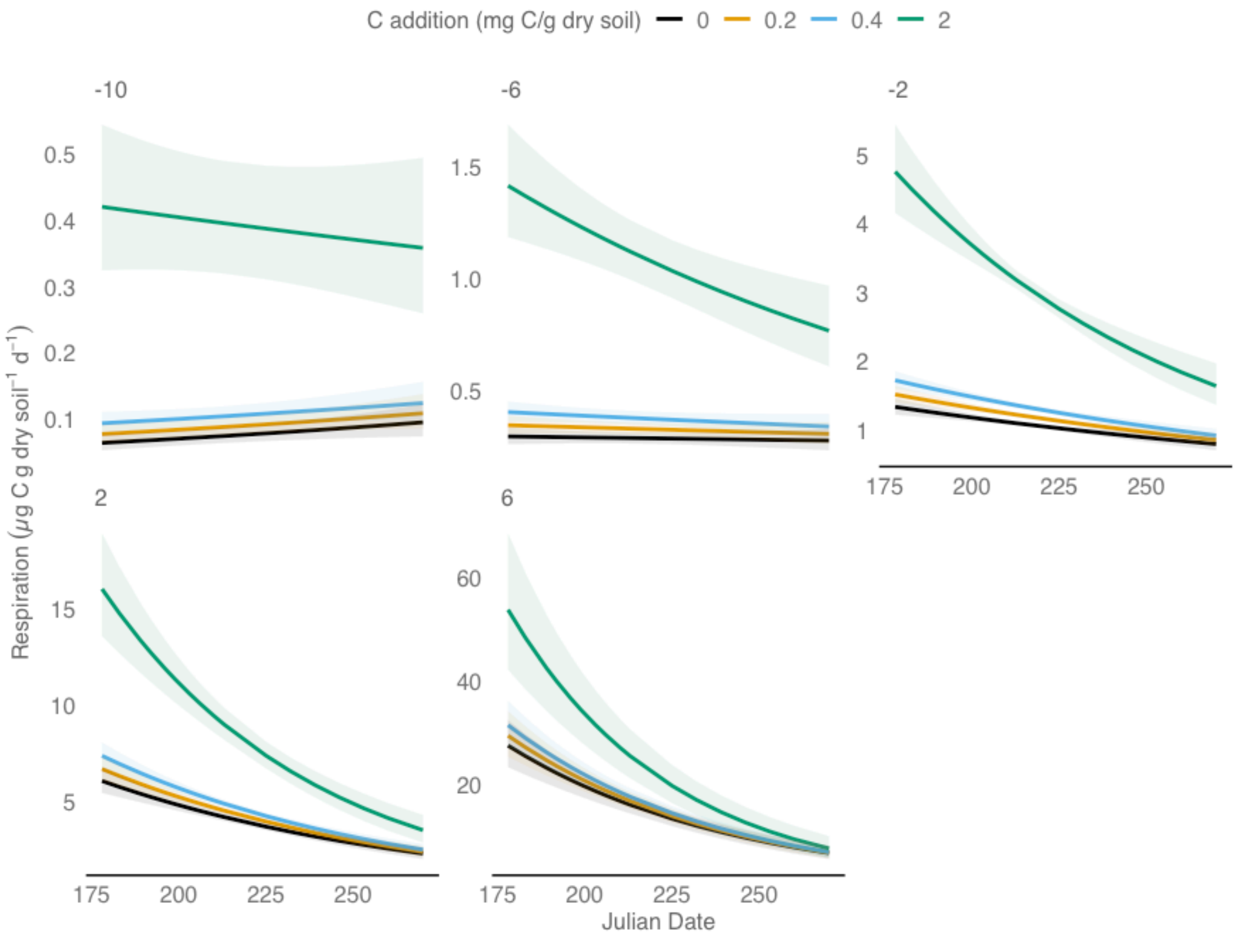

Supplementary Figure 5. Microbial respiration over time in the laboratory. Each panel shows linear mixed effects model predictions of the effects of labile C addition and time on microbial respiration at a given soil temperature (n=4 samples/temperature*C addition treatment). Shading indicates the 95% confidence intervals on the predictions.

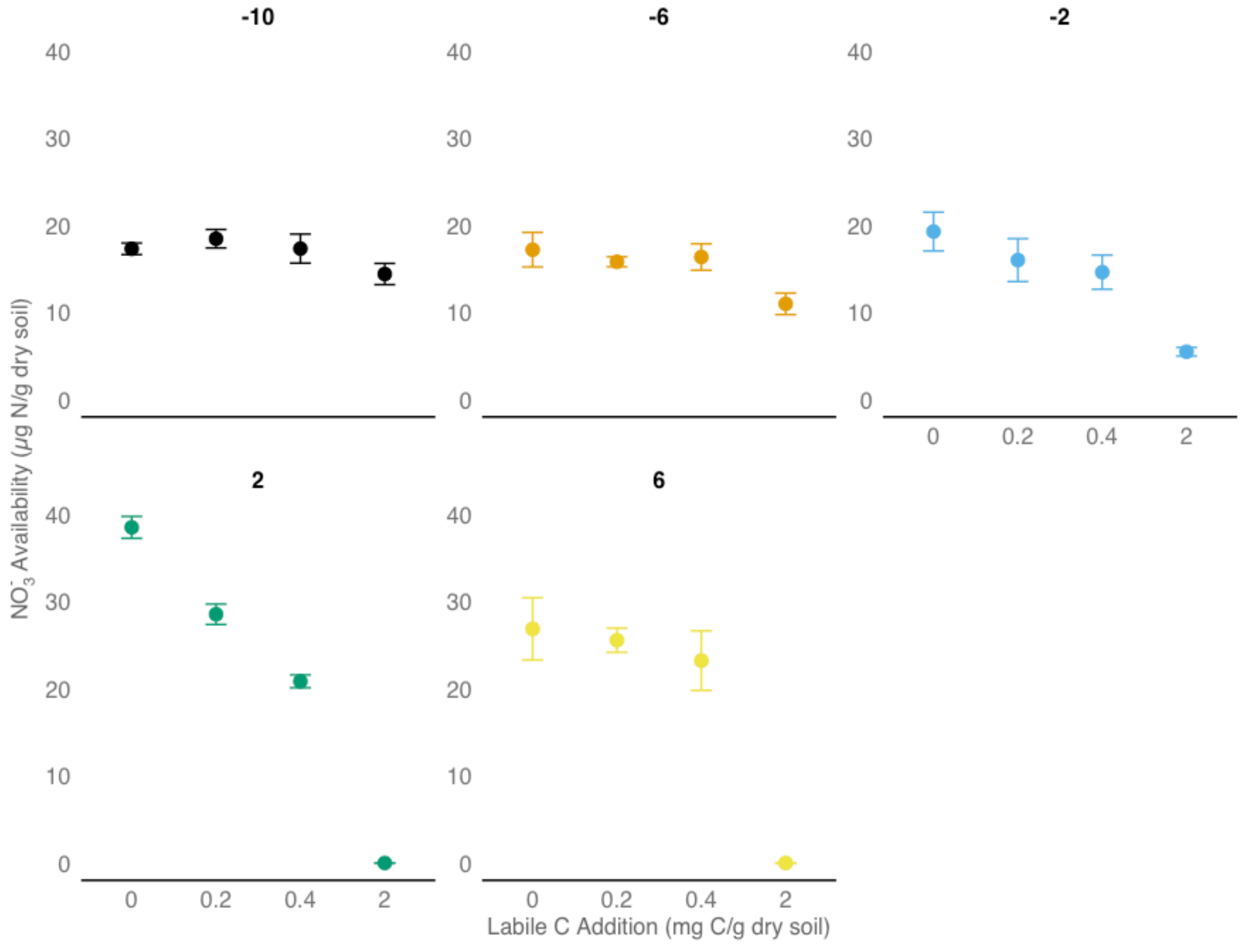

Supplementary Figure 6. Temperature and labile C addition effects on NO_3^- availability. Laboratory incubations were performed over three months using homogenized composite organic soils collected from our hydric and xeric sites (n=4 samples/temperature*C addition treatment). Bars are ± 1.0 S.E.M.

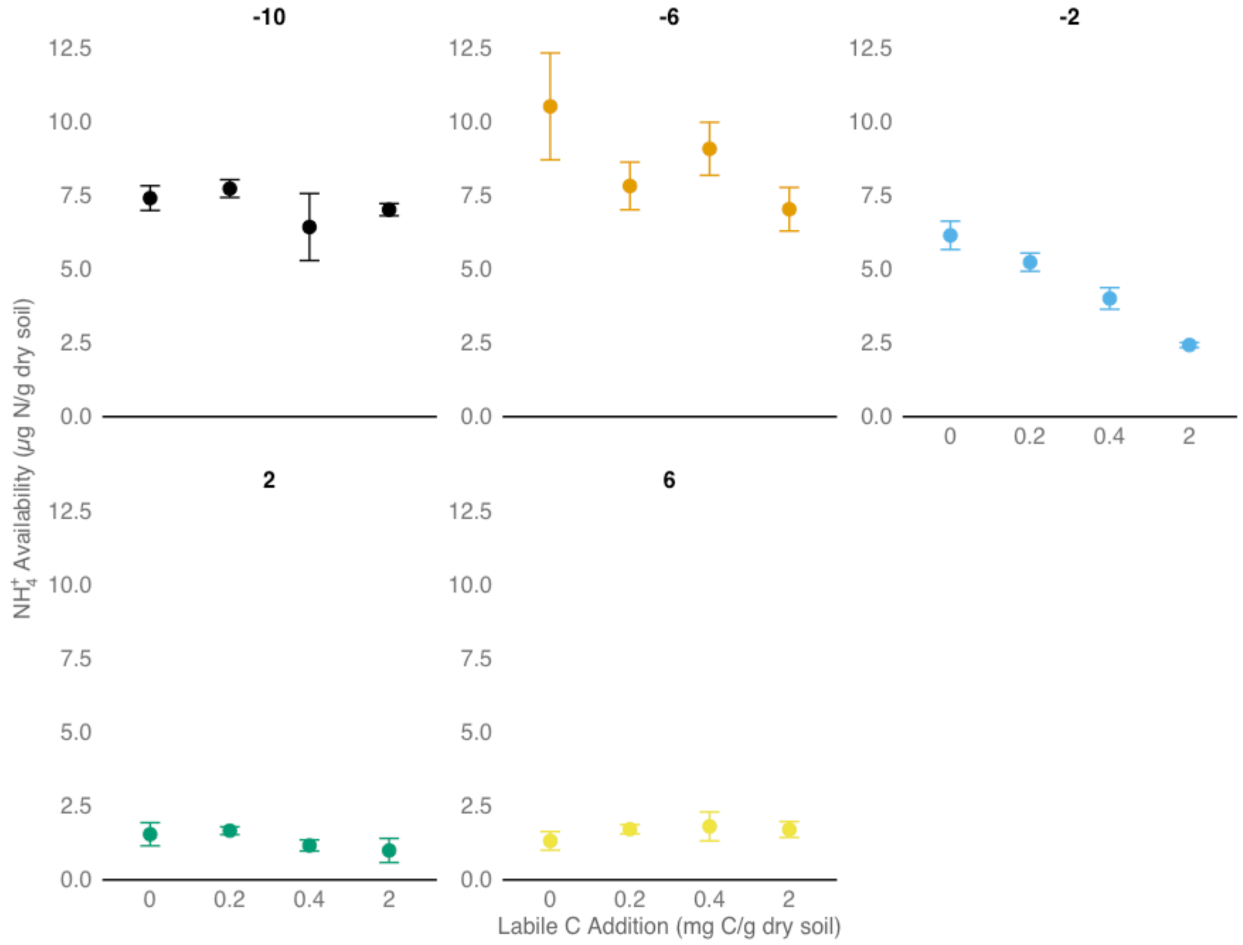

Supplementary Figure 7. Temperature and labile C addition effects on NH_4^+ availability. Laboratory incubations were performed over three months using homogenized composite organic soils collected from our hydric and xeric sites (n=4 samples/temperature*C addition treatment). Bars are ± 1.0 S.E.M.

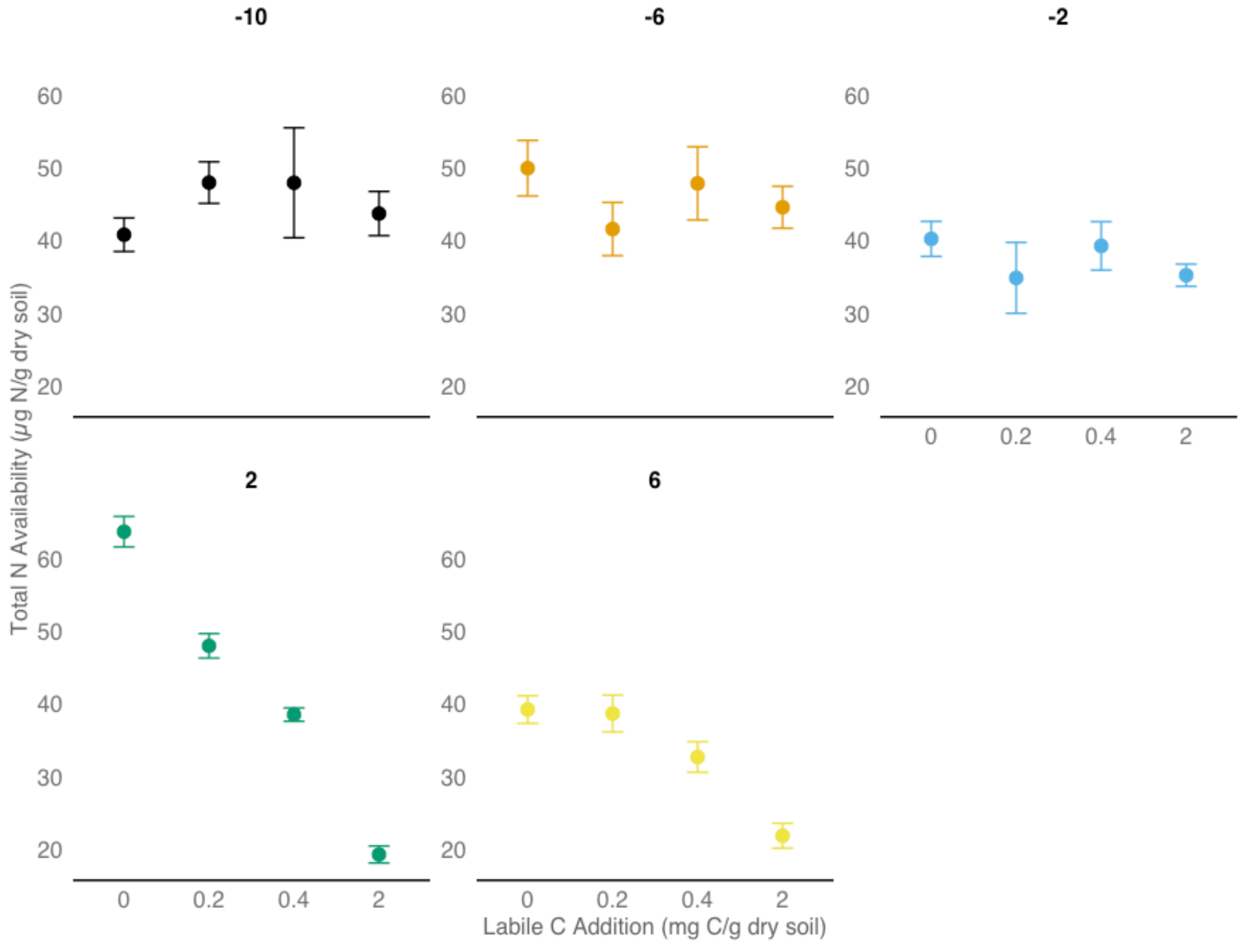

Supplementary Figure 8. Temperature and labile C addition effects on total nitrogen (N) availability. Laboratory incubations were performed over three months using homogenized composite organic soils collected from our hydric and xeric sites (n=4 samples/temperature*C addition treatment). Bars are ± 1.0 S.E.M.

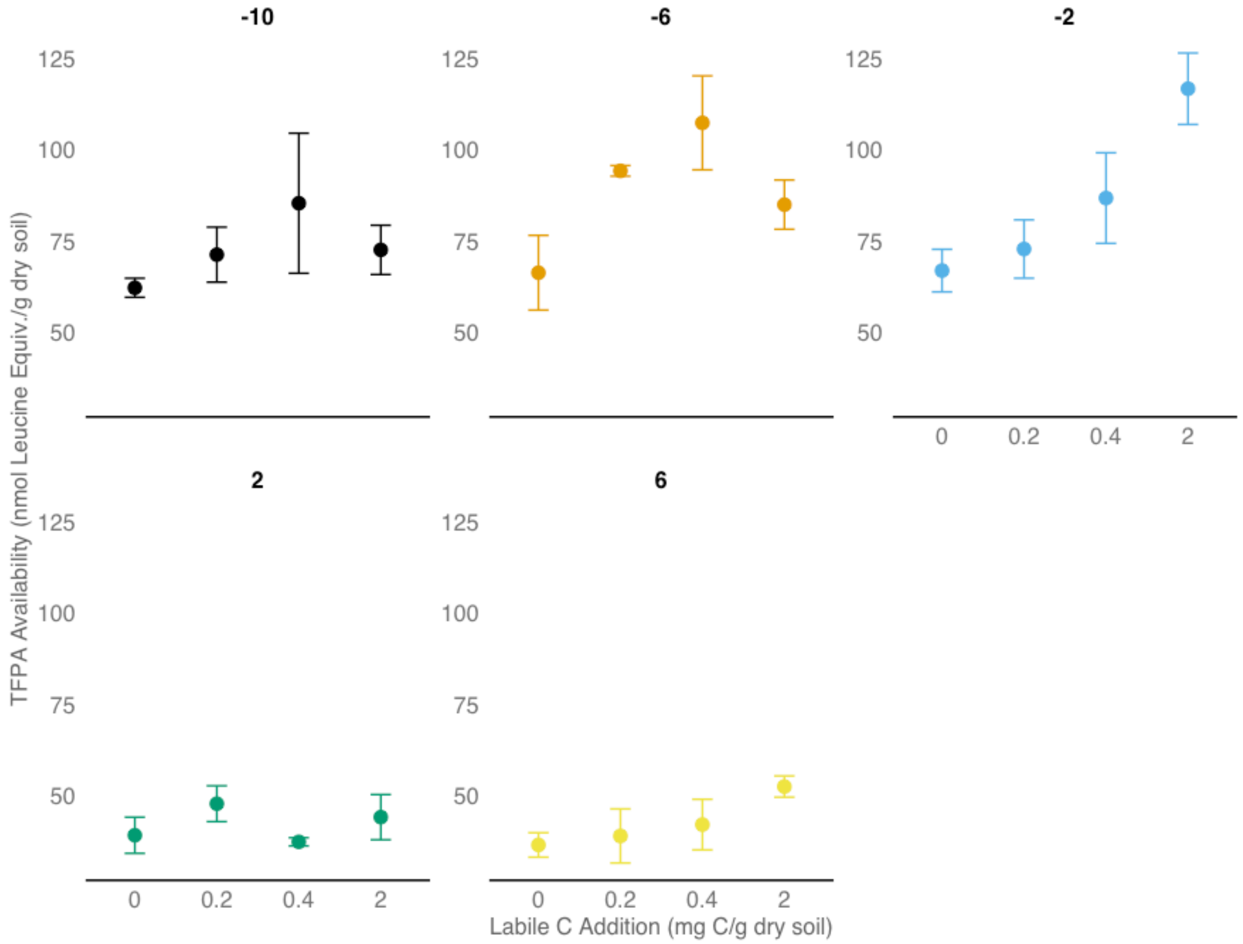

Supplementary Figure 9. Temperature and labile C addition effects on total free primary amine (TFPA) availability. TFPA is an indicator of amino acid availability. Laboratory incubations were performed over three months using homogenized composite organic soils collected from our hydric and xeric sites (n=4 samples/temperature*C addition treatment). Bars are ± 1.0 S.E.M.

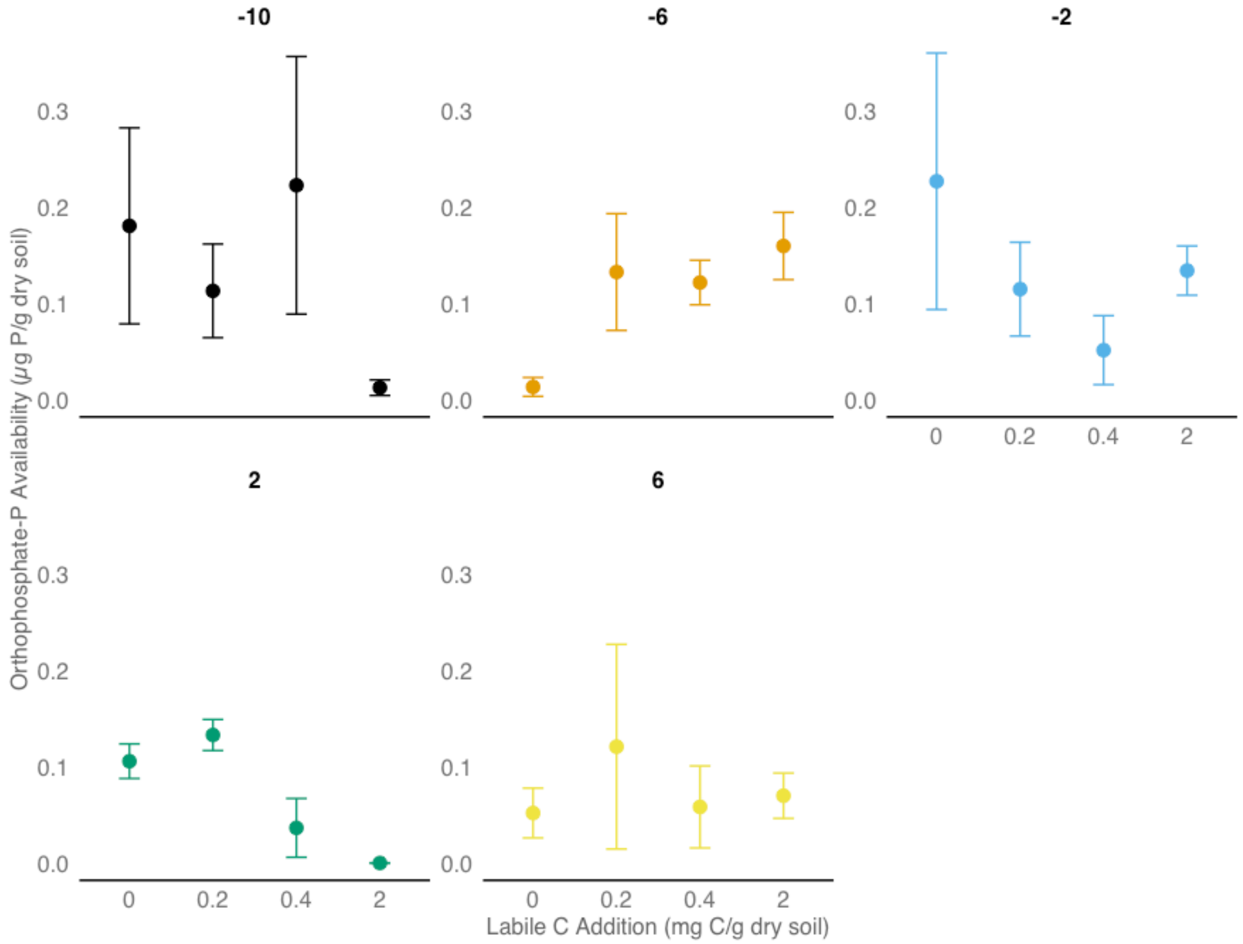

Supplementary Figure 10. Temperature and labile C addition effects on Orthophosphate-P availability. Laboratory incubations were performed over three months using homogenized composite organic soils collected from our hydric and xeric sites (n=4 samples/temperature*C addition treatment). Bars are ± 1.0 S.E.M.

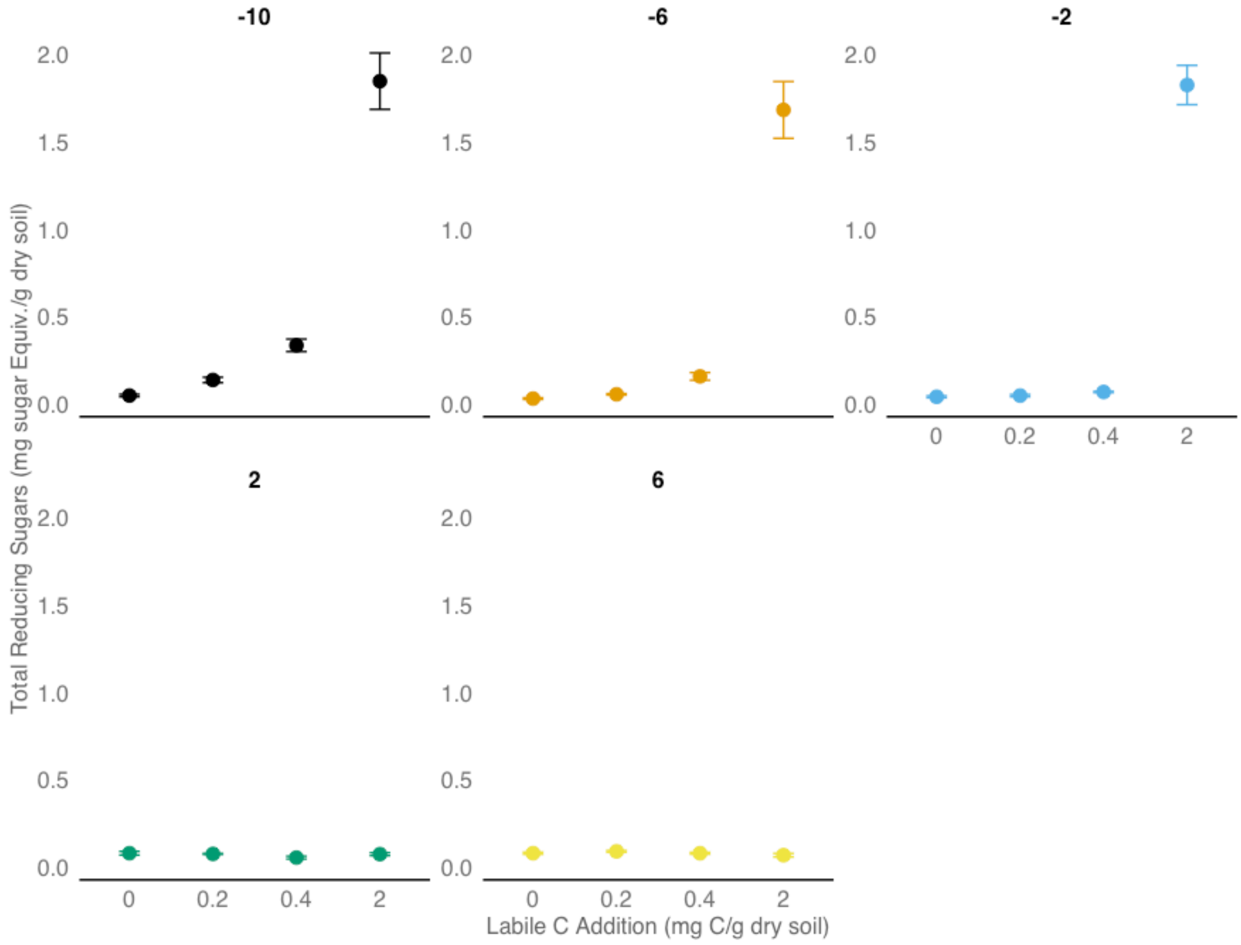

Supplementary Figure 11. Temperature and labile C addition effects on total reducing sugar (TRS) concentrations. Labile C additions were made using cellobiose, which is a reducing sugar. TRS assays reveal increasing microbial utilization of the added labile C with increasing temperature, including temperature increases below 0°C. Laboratory incubations were performed over three months using homogenized composite organic soils collected from our hydric and xeric sites (n=4 samples/temperature*C addition treatment). Bars are ± 1.0 S.E.M.

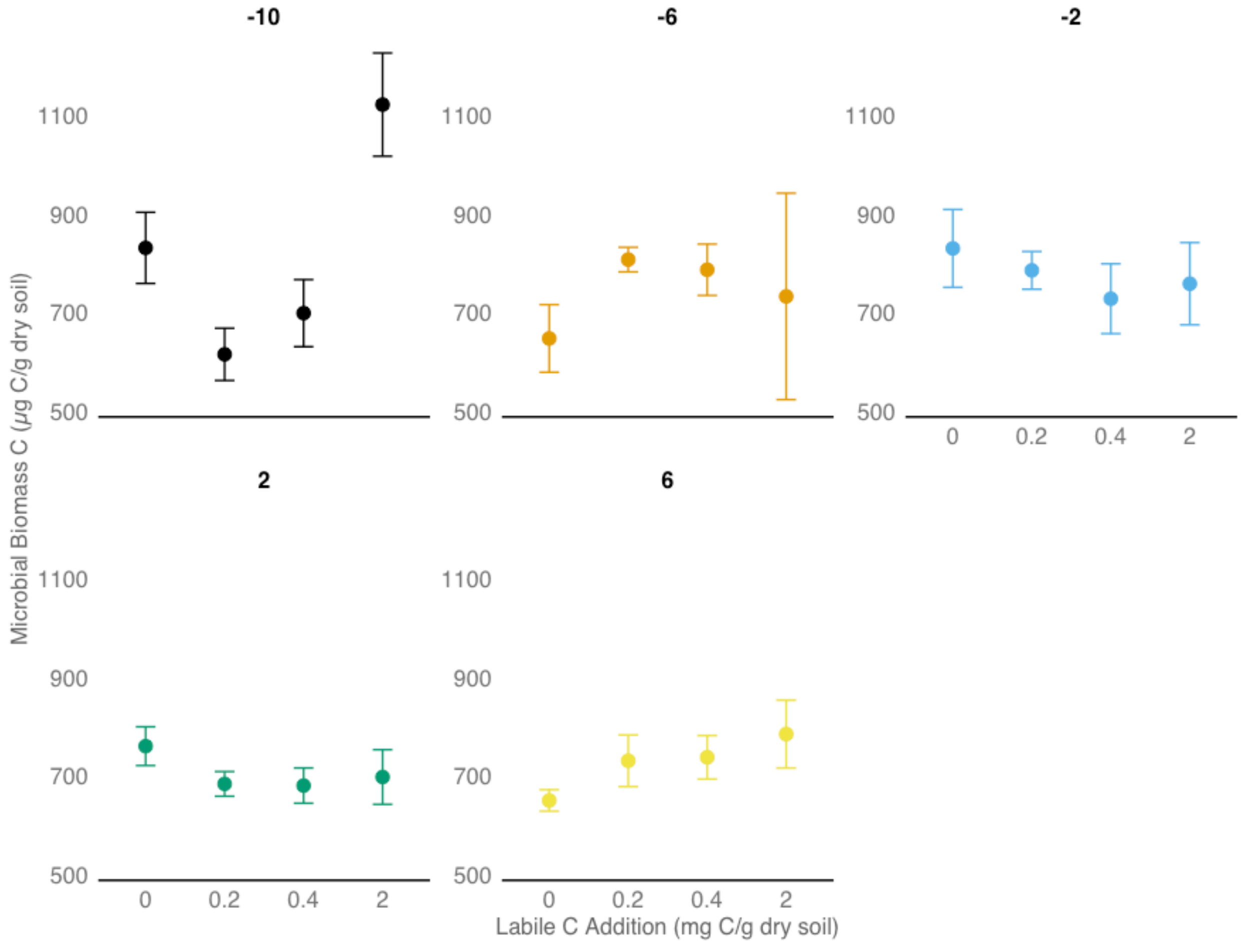

Supplementary Figure 12. Temperature and labile C addition effects on microbial biomass carbon (C). Laboratory incubations were performed over three months using homogenized composite organic soils collected from our hydric and xeric sites (n=4 samples/temperature*C addition treatment). Bars are ± 1.0 S.E.M.

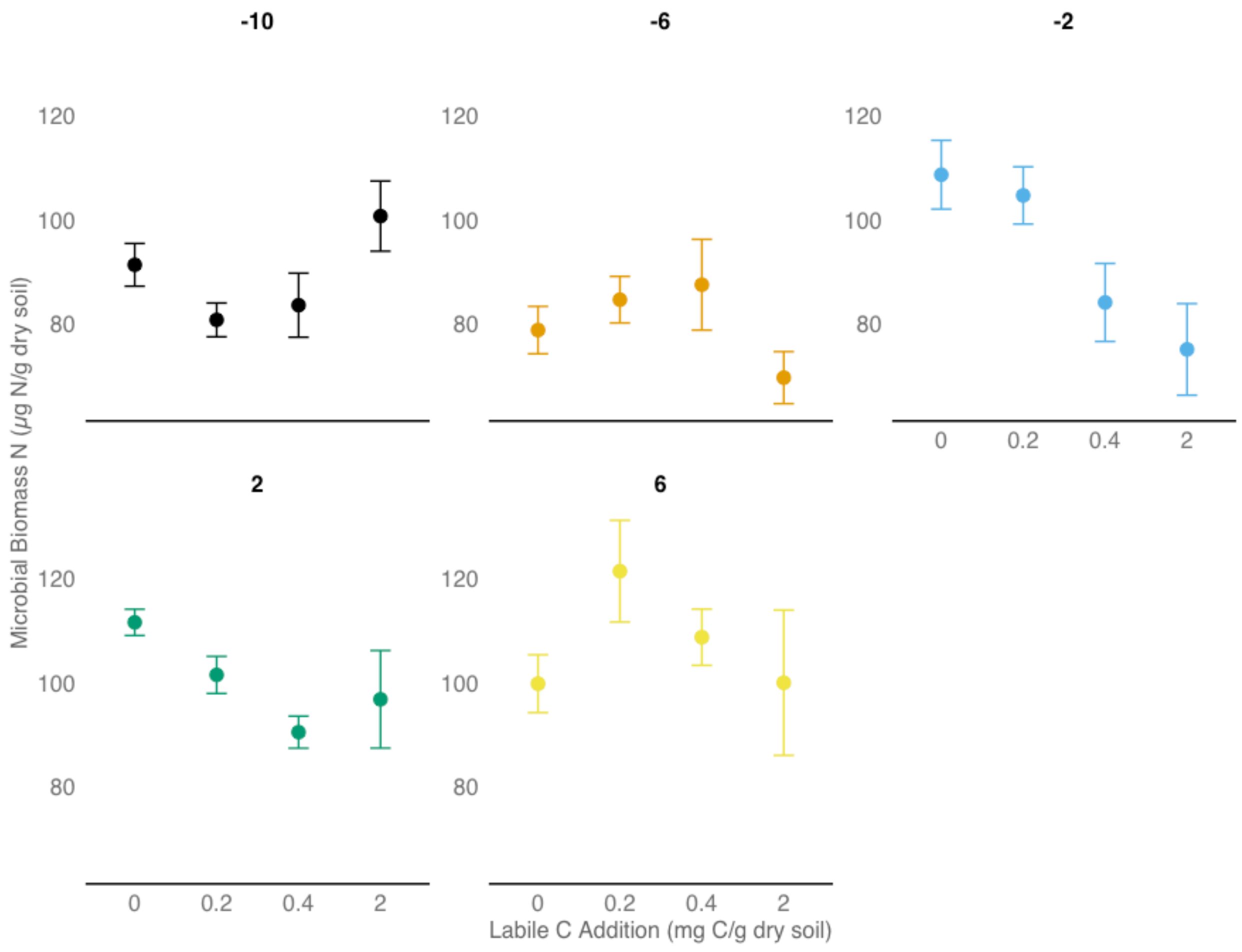

Supplementary Figure 13. Temperature and labile C addition effects on microbial biomass nitrogen (N). Laboratory incubations were performed over three months using homogenized composite organic soils collected from our hydric and xeric sites (n=4 samples/temperature*C addition treatment). Bars are ± 1.0 S.E.M.

Supplementary Table 1. Soil C and N concentration (% dry mass) by site. Soils were collected from a depth interval of 8-12 cm during the 2018 field season. The soils were dried, homogenized and subsampled for analysis (n=4/site).

**Site**	**%C**	**%N**
Hydric	32.5	1.69
Mesic	33.1	1.49
Xeric	16.1	1.09
