# Supplementary material for: Labile carbon limits late winter microbial activity near Arctic treeline
Source: Nat Commun. 2020 Aug 12;11:4024. doi: 10.1038/s41467-020-17790-5 (PMC7423931; doi:10.1038/s41467-020-17790-5)
Supplement: Supplementary file 3 — Reporting Summary [file 41467_2020_17790_MOESM3_ESM.pdf]

nature research

Corresponding author(s): Patrick Sullivan

Last updated by author(s): Jul 14, 2020

Reporting Summary

Nature Research wishes to improve the reproducibility of the work that we publish. This form provides structure for consistency and transparency in reporting. For further information on Nature Research policies, see Authors & Referees and the Editorial Policy Checklist.

Statistics

For all statistical analyses, confirm that the following items are present in the figure legend, table legend, main text, or Methods section.

n/a	Confirmed	
☐	☒	The exact sample size (n) for each experimental group/condition, given as a discrete number and unit of measurement
☐	☒	A statement on whether measurements were taken from distinct samples or whether the same sample was measured repeatedly
☐	☒	The statistical test(s) used AND whether they are one- or two-sided<br>*Only common tests should be described solely by name; describe more complex techniques in the Methods section.*
☐	☒	A description of all covariates tested
☐	☒	A description of any assumptions or corrections, such as tests of normality and adjustment for multiple comparisons
☐	☒	A full description of the statistical parameters including central tendency (e.g. means) or other basic estimates (e.g. regression coefficient) AND variation (e.g. standard deviation) or associated estimates of uncertainty (e.g. confidence intervals)
☐	☒	For null hypothesis testing, the test statistic (e.g. F, t, r) with confidence intervals, effect sizes, degrees of freedom and P value noted<br>*Give P values as exact values whenever suitable.*
☒	☐	For Bayesian analysis, information on the choice of priors and Markov chain Monte Carlo settings
☒	☐	For hierarchical and complex designs, identification of the appropriate level for tests and full reporting of outcomes
☒	☐	Estimates of effect sizes (e.g. Cohen's d, Pearson's r), indicating how they were calculated

Our web collection on statistics for biologists contains articles on many of the points above.

Software and code

Policy information about availability of computer code

Data collection	Neither software, nor computer code were utilized during data collection.
Data analysis	Scripts written using R version 4.0.2 to analyze the data and produce the graphics have been archived along with each of the datasets in the Arctic Data Center of the National Science Foundation, USA: https://doi.org/10.18739/A2V40K067. The archived R scripts utilize the following R packages: propogate version 1.0-6, nlme version 3.1-148, interactions version 1.1.3 and ggplot2 version 3.3.1.

For manuscripts utilizing custom algorithms or software that are central to the research but not yet described in published literature, software must be made available to editors/reviewers. We strongly encourage code deposition in a community repository (e.g. GitHub). See the Nature Research guidelines for submitting code & software for further information.

Data

Policy information about availability of data

All manuscripts must include a data availability statement. This statement should provide the following information, where applicable:
- Accession codes, unique identifiers, or web links for publicly available datasets
- A list of figures that have associated raw data
- A description of any restrictions on data availability

Data presented in this article have been archived in the Arctic Data Center of the National Science Foundation, USA: https://doi.org/10.18739/A2V40K067. Long-term air temperature data for Kotzebue, Alaska are available from the National Center for Environmental Information at the National Oceanic and Atmospheric Administration (https://www.ncdc.noaa.gov/). Long-term precipitation data for Kotzebue are available from the Alaska Climate Research Center at the University of Alaska Fairbanks (http://akclimate.org/).

Field-specific reporting

Please select the one below that is the best fit for your research. If you are not sure, read the appropriate sections before making your selection.

☐ Life sciences ☐ Behavioural & social sciences ☒ Ecological, evolutionary & environmental sciences

For a reference copy of the document with all sections, see nature.com/documents/nr-reporting-summary-flat.pdf

Ecological, evolutionary & environmental sciences study design

All studies must disclose on these points even when the disclosure is negative.

Study description
The study used field observations, field experiments and temperature-controlled laboratory incubations to reveal that soil microbial communities can become limited by labile carbon availability during late winter near the Arctic treeline.

Research sample
The study examined inter-annual variation in late winter CO2 efflux from the soils surrounding 48 study trees distributed across 3 treeline sites (hydric, mesic, xeric) and 2 treatments (control and snowfence). Field glucose additions were made to 5 treatment plots, which were paired with 5 control plots, at each of the 3 treeline sites. Laboratory incubations were performed using 4 sub-samples of homogenized field soils for each combination of incubation temperature and labile carbon addition. The sample sizes were selected based upon the combination of logistical constraints and our past experience working in these ecosystems.

Sampling strategy
Sampling involved automated measurements made by micrometeorological stations and soil temperature sensors, field measurements of CO2 flux, and laboratory measurements of microbial respiration and soil nutrient availability. Sample sizes were determined by a combination of logistical constraints (e.g., how many field plots can be measured during a day in the Arctic winter) and past experience making these measurements at similar field sites with similar soils (e.g., Sullivan 2010). A composite homogenized soil was used for the laboratory incubations to minimize the role of sample-to-sample or plot-to-plot variation, which was captured in the field measurements, and to isolate the effects of carbon availability and temperature.

Data collection
Field measurements were recorded in a field book by Sullivan and Stokes. Sullivan performed computer data entry. McMillan recorded the laboratory data in digital format.

Timing and spatial scale
Field observational measurements were made in the last week of March in 2017, 2018 and 2019. The last week of March was selected for field sampling because this is when the seasonal snowpack is near its maximum depth. The field sites (Hydric, Mesic and Xeric) are 3-4 ha in size. The field glucose addition experiment was performed in the last week of March 2019. Field glucose additions were made to 1.0 m2 plots located in relatively homogeneous areas away from trees within the larger sites. Laboratory incubations were carried out from late June to late September of 2019.

Data exclusions
In the field observational study, a small number of sub-replicate measurements (n=4 of 432) showed exceptionally high subnivian CO2 concentrations, likely reflecting trapping of CO2 by a discontinuous ice layer within the snowpack or inadvertent probe penetration into the soil. These observations were excluded as extreme outliers from the dataset. We did not have pre-determined exclusion criteria. The two sub-replicate CO2 measurements that were excluded in 2018 had exceeded the 4000 ppm range of the analyzer. The range of the analyzer was increased before sampling in 2019. The two sub-replicate CO2 measurements that were excluded in 2019 were from the same plot as in 2018, were again greater than 4000 ppm, and were more than 1000 ppm higher than the next highest subnivean CO2 concentration.

Reproducibility
Our experimental studies were not repeated. The glucose addition experiment was carried out at three different sites and showed similar responses across sites. Respiration measurements in the laboratory incubation were made repeatedly over time on a total of 13 occasions. Respiration was below detection limits for 13 of 1072 observations. These observations were assigned a flux of 0.01 ug C g dry soil-1 day-1, which was consistent with the lowest detectable flux.

Randomization
The snowfence treatment was applied to every-other tree (spatially) at our treeline sites. The field glucose experiment was implemented along transects through representative and relatively homogeneous areas at each treeline site, with paired plots established every 6 m. The glucose treatment was randomly assigned to one plot in each pair. There was no need for random assignment of treatments in the laboratory incubation, as the experiment was conducted using homogenized soils.

Blinding
Blinding is not relevant when the subject is not conscious of the experiment.

Did the study involve field work? ☒ Yes ☐ No

Field work, collection and transport

Field conditions
Air temperature during March fieldwork in 2017, 2018 and 2019 ranged from -30 to -2 deg C. There were numerous instances of snowfall and care was taken to make sure subnivean CO2 concentrations were measured during prolonged periods of low wind speeds.

Location
Agashashok River, Noatak National Preserve, Northwest Alaska (67.47, -162.23)

Access and import/export
Our research in Noatak National Preserve is permitted by the National Park Service (Permit #: NOAT-2017-SCI-0001).

Disturbance	Soil sampling involved collection of soil cores to a depth of 15 cm. The associated ground disturbance was minimized by harvesting only the amount of soil required for the laboratory incubations. The logistics and costs of transporting frozen soils provided additional constraints.

Reporting for specific materials, systems and methods

We require information from authors about some types of materials, experimental systems and methods used in many studies. Here, indicate whether each material, system or method listed is relevant to your study. If you are not sure if a list item applies to your research, read the appropriate section before selecting a response.

Materials & experimental systems

n/a	Involved in the study
☒	☐ Antibodies
☒	☐ Eukaryotic cell lines
☒	☐ Palaeontology
☒	☐ Animals and other organisms
☒	☐ Human research participants
☒	☐ Clinical data

Methods

n/a	Involved in the study
☒	☐ ChIP-seq
☒	☐ Flow cytometry
☒	☐ MRI-based neuroimaging
