# Supplementary material for: Labile carbon limits late winter microbial activity near Arctic treeline
Source: Nat Commun. 2020 Aug 12;11:4024. doi: 10.1038/s41467-020-17790-5 (PMC7423931; doi:10.1038/s41467-020-17790-5)
Supplement: Supplementary file 2 — Peer Review File [file 41467_2020_17790_MOESM2_ESM.docx]

Peer Review Information

**Journal:** Nature Communications

**Manuscript title:** Labile carbon limits late winter microbial activity near Arctic treeline

**Corresponding author name(s):** Dr Patrick Sullivan

**Editorial notes:**

**Transferred manuscripts**

This manuscript has been previously reviewed at another journal that is not operating a transparent peer review scheme. This document only contains reviewer comments and rebuttal letters for versions considered at *Nature Communications*.

**Reviewer comments & decisions:**

**Reviewer comments, first version:**

Reviewer #1

The manuscript *Labile carbon limits late winter microbial activity near Arctic treeline* written by Sullivan and others addresses the important and topical issue of winter respiration in Arctic soils. The authors show, using field observations, an in-situ experiment, and laboratory incubations that microbes at their study sites are limited by labile carbon at the end of the winter season. While the methods section needs improvement and further methodological details might change my initial assessment, based on the available information, I think that the study is well designed and the methods and analyses employed by the authors are appropriate to answer their study questions.

To my knowledge, this is the first study to show definitively that end of winter soil microbial respiration is limited by labile C in Arctic soils. This is an important step forward for our understanding of the carbon balance of Arctic soils. However, it is my opinion that in order to be of interest to a broad audience, the authors would need to quantify the importance of this finding. In other words, the conclusions should be something like “we find that overwinter labile C limitation reduces CO2 loss by X% over the course of the winter, when compared to assuming no such limitation.” As it is written, the conclusions are interesting from a mechanistic perspective, but lack specifics about the broader implications to be appealing to a wide audience.

The idea that Arctic soils are limited by labile C over the winter has been inferred by previous studies dating back nearly two decades. See for example Grogan et al., 2001; Grogan & Jonasson, 2005; and Webb et al., 2016. Thus, the present study is an important addition to the literature because it confirms previous hypotheses, but is not an unqualified *discovery* (authors’ word choice in italics). The current version of this manuscript includes no mention of these previous studies and any future version **must** include a discussion of the previous work in this realm. In fact, perhaps this discussion could replace the introductory paragraph and current discussion of overwinter microbial activity in subalpine forests. It is much more relevant to discuss work done in C-rich permafrost soils.

The methods section needs more details, specifically the statistical analyses section. It would be particularly helpful to have the equations written out. I.e. instead of saying “using a nonlinear mixed effects model...,” and “model parameters were included for...,” the authors should specify which model was used and include the equation in the text so the reader can know how each explanatory variable was related to the response. For all equations, the authors should define all terms. For all models, the authors should report goodness of fit. Without that, the reader cannot evaluate how well the predictions made using the model might be.

Line-by-line comments to the authors

L27	Add oxford comma for clarity

L30	What is meant by “ultimate” here? The most important? I would say the most studied, yes, but there are many other controls that are known to affect soil microbial activity and I do not know of a study that orders them by importance, although see the Webb et al., 2016 supplement.

L30	Delete “are thought to”

L37	“microbial C limitation will reduce cold season CO2 emissions” in comparison to what?

L73	The speculation in this sentence is incorrect - spatial variation in substrate variability COULD address, at least in part, the potential for temporal development of labile C, as areas with high growing season productivity could be less limited in the winter than areas with low growing season productivity. Without testing if wintertime labile C limitation varies by growing season LAI and GPP, we don’t know.

L76	Split infinitive

L81	Add oxford comma for clarity

L83	Specify that the Brooks Range is in Alaska, USA

L85	Add oxford comma for clarity

L83-86 This sentence is awkwardly worded.

L86	Change to “cold air temperature**s**”

L92-95 Delete this sentence- not necessary. Additionally, unless the methods used were the same, it could be differences in methods causing the observed differences in CO2 flux.

L100 What time of year did the labile C additions occur?

L103	Delete “carefully (and completely)”

L105-107	Delete sentence on optimal timing of treatment response- unnecessary/move to methods

L131	Is it standard to report the t-value? I think the pvalue is what is most informative. Also, if you are reporting a t-value, does that mean these are based on t-tests? The methods section does not mention t-tests.

L144 Add oxford comma for clarity.

L162	In general, I think this is a weak ending to the paper. Nowhere else do you talk about shrub expansion and it isn’t immediately clear to me why that is the take-home message of your paper. If we are to assume that Arctic winter respiration is C limited across ecosystem types, why the focus on shrubs? Additionally, see DeMarco et al. 2014 (Ecology), whose findings suggest that snow trapping is unlikely to influence nutrient turnover enough to drive this positive feedback.

L174 I take issue with the phrase “our discovery;” see main comments.

L265 What was the total incubation time?

L297 Q10 needs to be defined and justification provided for it being used as the response variable.

L299 What is A and B and T and why is there not an equals sign?

Figure captions generally: avoid reproducing methods in figure captions

L346: Instead of the first sentence, would be good to remind the reader which year was the cold year. Also good to remind reader that these are field measurements in the title of the figure.

L348: Remove “contrasting”

L351: How do you show this statistically?
L358: Remind reader this is for field data

L485: Are these measurements or models? It would be nice to see some of the measurements rather than models.

References

Grogan, P., & Jonasson, S. (2005). Temperature and substrate controls on intra-annual variation in ecosystem respiration in two subarctic vegetation types. *Global Change Biology*, *11*(3), 465–475. <https://doi.org/10.1111/j.1365-2486.2005.00912.x>

Grogan, P., Illeris, L., Michelsen, A., & Jonasson, S. (2001). Respiration of recently-fixed plant carbon dominates mid-winter ecosystem CO2 production in sub-arctic heath tundra. *Climactic Change*, (50), 129–142.

Webb, E. E., Schuur, E. A. G., Natali, S. M., Oken, K. L., Bracho, R., Krapek, J. P., et al. (2016). Increased wintertime CO2 loss as a result of sustained tundra warming. *Journal of Geophysical Research: Biogeosciences*, 249–265. <https://doi.org/10.1002/2014JG002795.Received>

Reviewer #2 (Remarks to the Author):

Review of: Labile carbon limits late winter microbial activity near Arctic treeline

Manuscript number: NCOMMS-20-08501-T

General comments

This paper is very well-written, and I believe will be of broad interest to the soil science community.

The authors use a series of observational and experimental approaches (in the field, at the north western extent of the Alaskan treeline, and in the laboratory) to test how warmer winters influence microbial activity and nutrient cycling. They found that pronounced warming during Arctic winters could result in microbial depletion of labile carbon sources and reduced overwinter CO2 emissions to the atmosphere. This work is in-line with previous findings that winter CO2 production is an important component of total annual ecosystem respiration (~10%). However, their findings challenge recent Pan Arctic models that assume microbial respiration remains static over time. They suggest improved models will need to include temperature response curves that vary over time with fluctuations in substrate availability. Without these corrections, soil C losses from the Arctic may be overestimated.

Specific comments

Line 32: I would suggest moving the sentence “Rising air temperature…” up to line 30 so the abstract flows more smoothly.

Line 36: Throughout the manuscript ‘Arctic’ is typically capitalized but occasionally lowercase. I would capitalize throughout (see additionally lines 52, 58, 68, 145, 162, and 163).

Line 73: Remove the ‘/’ between and model

Line 99: In which year was the field experiment conducted (2019)?

Line 100: How much did the 100 g C/m2 increase average soil C levels by? Is it possible this amount of C induced a C fertilization effect?

Line 100: Is it possible the rate of glucose powder dissolution may vary between xeric and hydric sites (such that a single measurement at 72 hours is not an equal comparison)?

Line 103: At the test site in Anchorage (or at Agashashok) were CO2 emissions measured from pre-disturbed plots as well? These data are not necessary to correct CO2 flux from treatment plots (versus paired controls), but I think would be important to account for disturbance effects if the data were used for model development.

Line 107: Is the field experiment designed to show microbial communities are C-limited at the end of winter? I assume amending any soil with a large quantity of glucose will increase CO2 flux above a paired control (whether or not the site is C-limited). Without isotope tracing or a longer-term assessment of the consequences of that C addition (i.e. past 72 hours, or calculation of a rate change in CO2 production) I am not quite sure what this experiment is telling us. Contextualizing these results following line 113 would be very helpful.

Line 108: What was the a priori decision criteria to measure respiration after 72 hours (vs. sequential measurements that identify the absolute peak in respiration followed by a measured decline)?

Line 113: Do soil organic carbon pools differ significantly at the three ecotone sites? I think it would be helpful to include a table in supplementary information with background site information (soil moisture, C and N concentrations, etc). Figure 4 splits snow depth and CO2 flux by site, but it would be nice to see how the three sites differ in background conditions prior to experimental manipulation.

Line 115: Why was there a switch from amending soils with glucose (in the field) to cellobiose (in the lab)?

Line 129: Add a citation here.

Lines 138-143: Shifts in the balance between NO3- and NH4+ is very interesting! I know space is limited here, but there are lots of ideas thrown out without context or follow-up. Perhaps splitting this sentence into two would be helpful. I would suggest focusing on how this shift will influence i) microbial activity/gas flux and then ii) plant composition.

Lines 163-171: Feedbacks between the different vegetation communities at these sites and the composition/function of their underlying microbial communities is also very intriguing, especially as it relates to overwinter respiration and soil C persistence. For example, work by Julie Deslippe and others found that microbial community composition differs significantly between shrubs and sedges (where B. nana form ectomycorrhizal associations and E. vaginatum do not). Follow-up work found these communities have different substrate use efficiencies and extracellular enzyme activities that influence seasonal nutrient availability and microbial biomass (Lynch). The effect of these different substrate use efficiencies and/or activation of a microbial priming response would certainly influence C persistence if treelines continue their northward extension (Hartley).

Deslippe, J. R., Hartmann, M., Mohn, W. W., & Simard, S. W. (2011). Long‐
term experimental manipulation of climate alters the ectomycorrhizal
community of Betula nana in Arctic tundra. Global change biology, 17(4),
1625-1636.

Lynch, L. M., Machmuller, M. B., Cotrufo, M. F., Paul, E. A., & Wallenstein,
M. D. (2018). Tracking the fate of fresh carbon in the Arctic tundra: Will
shrub expansion alter responses of soil organic matter to warming?. Soil
Biology and Biochemistry, 120, 134-144.

Hartley, I. P., Garnett, M. H., Sommerkorn, M., Hopkins, D. W., Fletcher, B.
J., Sloan, V. L., ... & Wookey, P. A. (2012). A potential loss of carbon
associated with greater plant growth in the European Arctic. Nature Climate
Change, 2(12), 875-879.

Line 196: How frequently are the meteorological data recorded?

Line 215: Was the LiCor calibrated in-field? How long were the CO2 measurement collection periods?

Line 241: Power should be powder.

Line 267: Were soil weights monitored throughout the three-month experiment to ensure constant soil moisture?

Line 273: What was the range of the calibration curve?

Lines 291: What are the analytical detection limits for nitrate, ammonium, and orthophosphate-P?

Line 299: For clarity, please define A and B

Figure 3: Change the year legend to match the figure caption (2016/2017, etc). Can the equations and R2 values be reported as well?

Figure 4: Can the P-value be included? ‘Results show strong stimulation of CO2 efflux (P<0.01?), which is a proxy…’

Figures S4-S11: Consider adding letters to plots that identify statistically significant differences in the response variable across the different levels of labile C addition. Additionally, the last sentence in each figure caption refers to bars representing 1.0 S.E.M. Is this different than before (where bars represent S.E.M.)?

A little more contextualization of findings would be helpful, but overall I think this is a really exciting collection of studies.

**Author rebuttal, first version:**

Reviewer #1 (Remarks to the Author):

The manuscript Labile carbon limits late winter microbial activity near Arctic treeline written by Sullivan and others addresses the important and topical issue of winter respiration in Arctic soils. The authors show, using field observations, an in-situ experiment, and laboratory incubations that microbes at their study sites are limited by labile carbon at the end of the winter season. While the methods section needs improvement and further methodological details might change my initial assessment, based on the available information, I think that the study is well designed and the methods and analyses employed by the authors are appropriate to answer their study questions.

**We appreciate the reviewer’s positive assessment of our manuscript. We have added the requested details to our Methods section. Please see below for details.**

To my knowledge, this is the first study to show definitively that end of winter soil microbial respiration is limited by labile C in Arctic soils. This is an important step forward for our understanding of the carbon balance of Arctic soils. However, it is my opinion that in order to be of interest to a broad audience, the authors would need to quantify the importance of this finding. In other words, the conclusions should be something like “we find that overwinter labile C limitation reduces CO2 loss by X% over the course of the winter, when compared to assuming no such limitation.” As it is written, the conclusions are interesting from a mechanistic perspective, but lack specifics about the broader implications to be appealing to a wide audience.

**We agree with the reviewer that quantifying the magnitude of the labile C limitation effect on total winter CO_2_ efflux is a logical next step in this line of research, but it is unfortunately not possible with currently available data. At present labile C pool sizes are unknown and it is uncertain when during the winter labile C limitation might develop and at what rate. We recently deployed a system for continuous monitoring of winter CO_2_ efflux at our mesic treeline site and we hope to expand our continuous winter CO_2_ flux monitoring to include systems in snowfence and control plots at each of our three treeline sites. Results from 3-4 winters with (hopefully) contrasting temperature and snowpack regimes. This will allow us to identify when labile C limitation develops under contrasting scenarios of temperature, snowpack development and site productivity. If we were to attempt to quantify the effect of labile C limitation on total winter CO_2_ efflux now, we would be forced to make some questionable assumptions. Nonetheless, as the reviewer notes, our study is the first to show definitively that end of winter soil microbial respiration can be limited by labile C in Arctic soils. We were also able to show the potential implications of labile C limitation for soil nutrient cycling.**

The idea that Arctic soils are limited by labile C over the winter has been inferred by previous studies dating back nearly two decades. See for example Grogan et al., 2001; Grogan & Jonasson, 2005; and Webb et al., 2016. Thus, the present study is an important addition to the literature because it confirms previous hypotheses, but is not an unqualified discovery (authors’ word choice in italics). The current version of this manuscript includes no mention of these previous studies and any future version must include a discussion of the previous work in this realm. In fact, perhaps this discussion could replace the introductory paragraph and current discussion of overwinter microbial activity in subalpine forests. It is much more relevant to discuss work done in C-rich permafrost soils.

**We are thankful to the reviewer for reminding us of these earlier studies. They provide an excellent set-up for our study and they aid in formulation of hypotheses regarding the likely spatial extent of overwinter microbial labile C limitation. We have incorporated all three of these references in a revised version of our introduction, which now highlights the development of the field over time and the current gaps in our understanding.**

The methods section needs more details, specifically the statistical analyses section. It would be particularly helpful to have the equations written out. I.e. instead of saying “using a nonlinear mixed effects model…,” and “model parameters were included for…,” the authors should specify which model was used and include the equation in the text so the reader can know how each explanatory variable was related to the response. For all equations, the authors should define all terms. For all models, the authors should report goodness of fit. Without that, the reader cannot evaluate how well the predictions made using the model might be.

**We have included the model formulas, as requested. We have also included the traditional names for our analyses, such as analysis of variance (ANOVA), analysis of covariance (ANCOVA) and multiple regression. Finally, we have included goodness of fit statistics for each of the models.**

Line-by-line comments to the authors

L27 Add oxford comma for clarity

**Done**

L30 What is meant by “ultimate” here? The most important? I would say the most studied, yes, but there are many other controls that are known to affect soil microbial activity and I do not know of a study that orders them by importance, although see the Webb et al., 2016 supplement.

**This sentence was deleted when revising the abstract.**

L30 Delete “are thought to”

**We think it is important to retain this “softener”, because the magnitudes of these effects are still being worked out. Our results suggest the effect of warmer winter soils on CO_2_ efflux may not be as large as previously thought, while the reviewer points out below that the effect of warmer winter soils on soil nutrient availability to plants may similarly not be as large as previously suggested.**

L37 “microbial C limitation will reduce cold season CO2 emissions” in comparison to what?

**In comparison with CO_2_ emissions in the absence of microbial C limitation. The abstract is limited to 150 words, so there is limited space to elaborate. However, we have added “expected” to indicate that microbial labile C limitation will reduce “expected” CO_2_ emissions under warming.**

L73 The speculation in this sentence is incorrect - spatial variation in substrate variability COULD address, at least in part, the potential for temporal development of labile C, as areas with high growing season productivity could be less limited in the winter than areas with low growing season productivity. Without testing if wintertime labile C limitation varies by growing season LAI and GPP, we don’t know.

**We agree and have softened this sentence by inserting “might not” in the place of “does not” to better reflect the uncertainty.**

L76 Split infinitive

**We have deleted “of this revision”.**

L81 Add oxford comma for clarity

**Done**

L83 Specify that the Brooks Range is in Alaska, USA

**Done**

L85 Add oxford comma for clarity

**Done**

L83-86 This sentence is awkwardly worded.

**We moved the latter half of this sentence, which briefly described the field sites, up to the end of the Introduction.**

L86 Change to “cold air temperatures”

**Done**

L92-95 Delete this sentence- not necessary. Additionally, unless the methods used were the same, it could be differences in methods causing the observed differences in CO2 flux.

**The methods are the same for the Agashashok and should be comparable with Niwot, as both were based on measurements of subnivean CO_2_ concentrations and estimates of diffusion through the snowpack. We have made the comparability of the methods clear in the text.**

L100 What time of year did the labile C additions occur?

**Late March. It was in the figure caption, but we have added it to this location in the text as well.**

L103 Delete “carefully (and completely)”

**We have removed “carefully” because that should be a given in science. We have retained completely, however.**

L105-107 Delete sentence on optimal timing of treatment response- unnecessary/move to methods

**We would like to leave this sentence in the main text, as it briefly describes the preliminary glucose addition experiment in Anchorage, while also justifying our decision to measure the response at 72 hours.**

L131 Is it standard to report the t-value? I think the pvalue is what is most informative. Also, if you are reporting a t-value, does that mean these are based on t-tests? The methods section does not mention t-tests.

**This is a multiple regression analysis, which we have clarified in the Methods. T is the appropriate test statistic.**

L144 Add oxford comma for clarity.

**Done, but note that this sentence has been moved to the top of the Discussion.**

L162 In general, I think this is a weak ending to the paper. Nowhere else do you talk about shrub expansion and it isn’t immediately clear to me why that is the take-home message of your paper. If we are to assume that Arctic winter respiration is C limited across ecosystem types, why the focus on shrubs? Additionally, see DeMarco et al. 2014 (Ecology), whose findings suggest that snow trapping is unlikely to influence nutrient turnover enough to drive this positive feedback.

**We agree and have revised the concluding paragraph to focus on future directions, which reflects our belief that this paper will serve as a catalyst for further research on microbial labile C limitation in the Arctic and beyond.**

L174 I take issue with the phrase “our discovery;” see main comments.

**Sorry. We have replaced “discovery” with “finding” in the second sentence of the Discussion. We removed it from the final sentence of the Discussion when revising the final paragraph.**

L265 What was the total incubation time?

**Three months. We have added that information.**

L297 Q10 needs to be defined and justification provided for it being used as the response variable.

**We don’t actually report Q_10_ values in the manuscript, nor do we use them as a response variable in our statistical analyses. We simply use a Q_10_ temperature response model (an exponential model that includes the Q_10_ value as a term in the model) to describe the form of the relationship between temperature and either respiration or CO_2_ efflux. We have added the definition of Q_10_ to the Methods, where we show the form of the Q_10_ temperature response model.**

L299 What is A and B and T and why is there not an equals sign? Figure captions generally: avoid reproducing methods in figure captions

**We have added “CO_2_ Flux=” to the left-hand side of the equation and we have defined A, B and T. We are going to defer to the editors regarding the amount of information contained in the figure captions. Many readers only read the abstract and then look at the figures. Informative figure captions can be useful for readers who only have time to “skim” the paper.**

L346: Instead of the first sentence, would be good to remind the reader which year was the cold year. Also good to remind reader that these are field measurements in the title of the figure.

**We agree and have edited the figure caption to remind the readers that 2016/2017 was the relatively cold winter, while 2017/2018 and 2018/2019 were the warm winters.**

L348: Remove “contrasting”

**Done**

L351: How do you show this statistically?

**We have added Monte Carlo simulations to estimate the 95% confidence intervals for Q_10_ model predictions for each late March sampling campaign at a common soil temperature of -3 deg C. This analysis shows clear separation between confidence intervals on the predictions for 2017 (at the end of the cold winter) and those for 2018 and 2019 (at the end of the warm winters).**

L358: Remind reader this is for field data

**We have added “in the field” to the first sentence of the figure caption.**

L485: Are these measurements or models? It would be nice to see some of the measurements rather than models.

**These are predictions (and associated standard errors) based on model fits to the data. We have made certain that this is clear in the figure caption. The measured data appear in Figures S5-S8.**

References

Grogan, P., & Jonasson, S. (2005). Temperature and substrate controls on intra-annual variation in ecosystem respiration in two subarctic vegetation types. Global Change Biology, 11(3), 465–475. <https://doi.org/10.1111/j.1365-2486.2005.00912.x>

Grogan, P., Illeris, L., Michelsen, A., & Jonasson, S. (2001). Respiration of recently-fixed plant carbon dominates mid-winter ecosystem CO2 production in sub-arctic heath tundra. Climactic Change, (50), 129–142.

Webb, E. E., Schuur, E. A. G., Natali, S. M., Oken, K. L., Bracho, R., Krapek, J. P., et al. (2016). Increased wintertime CO2 loss as a result of sustained tundra warming. Journal of Geophysical Research: Biogeosciences, 249–265. https://doi.org/10.1002/2014JG002795.Received

Reviewer #2 (Remarks to the Author):

Review of: Labile carbon limits late winter microbial activity near Arctic treeline

Manuscript number: NCOMMS-20-08501-T

General comments

This paper is very well-written, and I believe will be of broad interest to the soil science community.

The authors use a series of observational and experimental approaches (in the field, at the north western extent of the Alaskan treeline, and in the laboratory) to test how warmer winters influence microbial activity and nutrient cycling. They found that pronounced warming during Arctic winters could result in microbial depletion of labile carbon sources and reduced overwinter CO2 emissions to the atmosphere. This work is in-line with previous findings that winter CO2 production is an important component of total annual ecosystem respiration (~10%). However, their findings challenge recent Pan Arctic models that assume microbial respiration remains static over time. They suggest improved models will need to include temperature response curves that vary over time with fluctuations in substrate availability. Without these corrections, soil C losses from the Arctic may be overestimated.

Specific comments

Line 32: I would suggest moving the sentence “Rising air temperature…” up to line 30 so the abstract flows more smoothly.

**Good suggestion. We have made this change.**

Line 36: Throughout the manuscript ‘Arctic’ is typically capitalized but occasionally lowercase. I would capitalize throughout (see additionally lines 52, 58, 68, 145, 162, and 163).

**We have gone ahead and capitalized “Arctic” throughout, although we recognize this is ultimately a decision for the copy editors.**

Line 73: Remove the ‘/’ between and model

**That was a typo. Thanks. It was supposed to be “and/or” and has been changed accordingly.**

Line 99: In which year was the field experiment conducted (2019)?

**Yes. 2019. That is now clear in the text.**

Line 100: How much did the 100 g C/m2 increase average soil C levels by? Is it possible this amount of C induced a C fertilization effect?

**We don’t have total soil C stock estimates for our sites. Estimates from Imnavait Creek on the North Slope of the Brooks Range are ~16 kg C/m2 in wet sedge, ~11 kg C/m2 in tussock tundra and ~12 kg C/m2 in dry heath (Euskirchen et al. 2017, Ecosystems 20: 960-974). We assume that the reviewer is wondering if our glucose additions may have stimulated microbial respiration of older soil organic matter (via “priming”). That is possible. However, the added 100 g C/m2 would have been enough to sustain a doubling of microbial respiration for far longer than our 72-hour experiment. Additionally, the Lynch et al. (2018) paper, which the reviewer mentions, found no evidence of priming in response to labile C additions to tussock and shrub tundra during the growing season. An isotopic study would be necessary to differentiate respiration of the added glucose, from respiration of recent plant inputs, from respiration of old soil organic matter. Such a tracer study was well-beyond the scope of our study, but would make for a nice study in the future.**

Line 100: Is it possible the rate of glucose powder dissolution may vary between xeric and hydric sites (such that a single measurement at 72 hours is not an equal comparison)?

**It is possible, although soil surface temperatures were fortuitously very similar across sites during the field glucose experiment. We were careful about drawing conclusions regarding the presence or absence of differences across sites in the response to glucose addition and instead focused on the main result that field glucose addition led to large increases in CO_2_ efflux irrespective of site.**

Line 103: At the test site in Anchorage (or at Agashashok) were CO2 emissions measured from pre-disturbed plots as well? These data are not necessary to correct CO2 flux from treatment plots (versus paired controls), but I think would be important to account for disturbance effects if the data were used for model development.

**Yes. We made pre-treatment (pre-disturbance) CO_2_ flux measurements from both control and C addition plots. The pre-treatment measurements were included in our ANCOVA analysis to account for the possibility of pre-treatment differences in CO_2_ flux across plots. Inclusion of the pre-treatment data in the model also allowed us to account for any disturbance effect associated with snowpack removal and return to the plots. Inclusion of the pre-treatment data in the ANCOVA is explained in the Methods section.**

Line 107: Is the field experiment designed to show microbial communities are C-limited at the end of winter? I assume amending any soil with a large quantity of glucose will increase CO2 flux above a paired control (whether or not the site is C-limited). Without isotope tracing or a longer-term assessment of the consequences of that C addition (i.e. past 72 hours, or calculation of a rate change in CO2 production) I am not quite sure what this experiment is telling us. Contextualizing these results following line 113 would be very helpful.

**Yes. That was the aim of the field experiment, which we now state more explicitly. The field glucose addition experiment, which was modeled after that of Brooks et al. (2004), demonstrates that soil microbial respiration was not simply temperature limited in these soils. To gain more mechanistic insight into C limitation to microbial activity in frozen tundra soils we carried out the laboratory incubations. Our findings are unusually solid because we used two approaches (each with its own set of limitations) and both yielded the same conclusion - that soil microbes can deplete the pool of available labile C over winter and become C limited when soils are sufficiently warm.**

Line 108: What was the a priori decision criteria to measure respiration after 72 hours (vs. sequential measurements that identify the absolute peak in respiration followed by a measured decline)?

**We decided to measure the response to the glucose additions after 72 hours, because our preliminary experiment in a boreal forest in Anchorage showed a large and easily detectable CO_2_ flux response at this time point. Logistics were also a consideration. The Brooks Range treeline sites are very remote and involve camping during late winter measurement campaigns. The 72-hour measurement allowed us to initiate the experiment sequentially at each site over a three-day period and then return to the sites in the same order for response measurements over the next three-day period. If we wanted to make daily measurements, we would have needed to restrict the experiment to one site, which would have limited the spatial extent of our inferences.**

Line 113: Do soil organic carbon pools differ significantly at the three ecotone sites? I think it would be helpful to include a table in supplementary information with background site information (soil moisture, C and N concentrations, etc). Figure 4 splits snow depth and CO2 flux by site, but it would be nice to see how the three sites differ in background conditions prior to experimental manipulation.

**A table with average soil carbon and nitrogen concentrations for each of our three field sites has been added to the supplemental materials. Values were comparable between the hydric and mesic sites, but soil carbon content was ~50% lower in the xeric site.**

Line 115: Why was there a switch from amending soils with glucose (in the field) to cellobiose (in the lab)?

**We used cellobiose because it is a slightly more complex C form. Microbes need to cleave this glucose dimer with the enzyme beta-glucosidase for uptake. Using cellobiose rather than glucose, allowed us to measure changes in the activity of this enzyme and determine if its production was upregulated during the incubation. Beta-glucosidase activities at the end of the incubation did not vary as a function of C addition or temperature and are not shown. These additional details have been added to the incubation methods text.**

Line 129: Add a citation here.

**Further explanation and relevant citations have been added to the text.**

Lines 138-143: Shifts in the balance between NO3- and NH4+ is very interesting! I know space is limited here, but there are lots of ideas thrown out without context or follow-up. Perhaps splitting this sentence into two would be helpful. I would suggest focusing on how this shift will influence i) microbial activity/gas flux and then ii) plant composition.

**Thanks. More explanatory details and clearer connections between C and N availability were added to this paragraph to provide better context, and the sentence the reviewer refers to here was simplified and clarified.**

Lines 163-171: Feedbacks between the different vegetation communities at these sites and the composition/function of their underlying microbial communities is also very intriguing, especially as it relates to overwinter respiration and soil C persistence. For example, work by Julie Deslippe and others found that microbial community composition differs significantly between shrubs and sedges (where B. nana form ectomycorrhizal associations and E. vaginatum do not). Follow-up work found these communities have different substrate use efficiencies and extracellular enzyme activities that influence seasonal nutrient availability and microbial biomass (Lynch). The effect of these different substrate use efficiencies and/or activation of a microbial priming response would certainly influence C persistence if treelines continue their northward extension (Hartley).

Deslippe, J. R., Hartmann, M., Mohn, W. W., & Simard, S. W. (2011). Long‐
term experimental manipulation of climate alters the ectomycorrhizal
community of Betula nana in Arctic tundra. Global change biology, 17(4),
1625-1636.

Lynch, L. M., Machmuller, M. B., Cotrufo, M. F., Paul, E. A., & Wallenstein,
M. D. (2018). Tracking the fate of fresh carbon in the Arctic tundra: Will
shrub expansion alter responses of soil organic matter to warming?. Soil
Biology and Biochemistry, 120, 134-144.

**Very intriguing indeed! We agree that soil respiration responses to winter warming must vary across the landscape with plant and microbial community composition and function. We now note this more clearly in the last paragraph of the discussion, though we do not have the data required to compare our findings directly to these papers and can only comment in general on this. Furthermore, the Lynch study’s finding of no priming effect with added labile C highlights the dependence of microbial respiration on labile C that we observed and we now also cite this paper at the end of the introduction.**

Hartley, I. P., Garnett, M. H., Sommerkorn, M., Hopkins, D. W., Fletcher, B.
J., Sloan, V. L., ... & Wookey, P. A. (2012). A potential loss of carbon
associated with greater plant growth in the European Arctic. Nature Climate
Change, 2(12), 875-879.

**We also agree with the reviewer on the relevance of the Hartley et al. (2012) paper, which we have added to our revised Introduction.**

Line 196: How frequently are the meteorological data recorded?

**Hourly. iButtons recorded soil temperature at 4-hour intervals. We have added that information to the Methods.**

Line 215: Was the LiCor calibrated in-field? How long were the CO2 measurement collection periods?

**We have added this text to the Methods: “The zero of the LI-840 was checked daily before initiating field measurements, while regular measurements of atmospheric [CO_2_] provided an opportunity to check for analyzer drift. Readings of subnivean [CO_2_] consistently stabilized and were recorded within 1 minute of probe insertion.”**

Line 241: Power should be powder.

**Fixed. Thanks for catching that typo!**

Line 267: Were soil weights monitored throughout the three-month experiment to ensure constant soil moisture?

**Yes. This detail was added in the paragraph describing the respiration measurements.**

Line 273: What was the range of the calibration curve?

**This was already in the text, but we changed the wording to make it clearer and more obvious.**

Lines 291: What are the analytical detection limits for nitrate, ammonium, and orthophosphate-P?

**Added to methods text for all analytes: 0.01 µg PO4-P mL-1, 0.1 µg NO3-N mL-1, 0.1 µg NH4-N mL-1**

Line 299: For clarity, please define A and B

**Done. The other reviewer asked for this as well.**

Figure 3: Change the year legend to match the figure caption (2016/2017, etc). Can the equations and R2 values be reported as well?

**As it stands, the legend indicates the year in which the late March measurements were made. We think that switching to 2016/2017, for instance, could introduce confusion as to when the field measurements were made.**

**We would prefer not to report the equations for the Q_10_ curves in Figure 3, because the main point of our paper is that each represents just a snapshot in time and that the use of static Q_10_ temperature response functions is problematic. We worry that reporting the equations could imply otherwise. However, all the underlying data are available in the Arctic Data Center, where interested readers can download and use as they see fit.**

Figure 4: Can the P-value be included? ‘Results show strong stimulation of CO2 efflux (P<0.01?), which is a proxy…’

**Yes. This was reported in the Results section and has now been added to the figure caption as well.**

Figures S4-S11: Consider adding letters to plots that identify statistically significant differences in the response variable across the different levels of labile C addition. Additionally, the last sentence in each figure caption refers to bars representing 1.0 S.E.M. Is this different than before (where bars represent S.E.M.)?

**The data shown in these supplemental figures were analyzed using multiple regression, in which temperature and labile C addition were treated as continuous variables. In order to add letters indicating significant differences, the analyses would need to be re-done as ANOVAs, with temperature and labile C addition as categorical variables. A post-hoc test, like Tukey’s HSD, would then be necessary to test for differences among all possible pairs. We think that this dataset was better suited to analysis with a multiple regression approach, but we agree with the reviewer that bar graphs tend to evoke ANOVA in the minds of readers. We have re-done Figures S6-S13 using points rather than bars, because we think this will encourage readers to think of temperature and labile C addition as continuous variables. We have also revised the figure captions throughout, so that all read “Bars are 1.0 S.E.M.”.**

A little more contextualization of findings would be helpful, but overall I think this is a really exciting collection of studies.

**Reviewer comments, second version:**

Reviewer #1

Second review of *Labile carbon limits late winter microbial activity near Arctic treeline* by Sullivan and colleagues.

The authors have put a lot of work into thoroughly responding to reviewer comments, and the updated version is much improved as a result. However, there are still two main avenues for improvement.

The first is the statistical analyses. There are many claims in the text that are not backed up by statistical tests (or if they are, this is not made clear to the reader). As an example, L145 says “microbial respiratory responses to labile C addition [...] increased progressively with rising soil temperature (*statistical evidence needed*) [...]. Respiration, and the effect of C addition on respiration, declined during the three-month incubation (*statistical evidence needed*), especially at warmer temperatures (*statistical evidence needed*) [...].” I am not doubting that these trends exist, but I am arguing that they need to be substantiated.

I have noted other questions about the statistical analyses in the line-by-line comments.

The second is the Discussion. As written, it is pretty short, which I recognize is a feature of some *Nature Communications* papers. However, I think it could benefit from some discussion of the results of your nutrient analyses. As it is, a large chunk of that work is relegated to the results and supplementary material and the reader is left wondering about its broader significance. It is possible that these results could be woven into the existing discussion, or an additional paragraph could replace one of the existing.

Line-by-line comments:

L40-42 Is this sentence missing a word or punctuation? Perhaps it should read “...liquid water and solutes, which depress the freezing point and minimize the important physical constraints...”

L52	Update to “greatest increases in temperature have been...”

L56	change “show dramatic” to “indicate” because the regressions are only significant in one month, hardly dramatic.

L57	change to “The combination of increasing winter temperatures and precipitation will lead to...”

L59	do you mean in terms of the carbon cycle? Or what kinds of biological responses? Either way, should include some references here.

L77	remove “it was shown that” and update to read “...birch trees stimulated microbial respiration...”

L82	remove “it was shown that”

L105 In L119 and L137, you give a brief overview of what you did before delving into the results. Something similar would be helpful here; before this first sentence, you don’t even mention that measurements were taken for 3 winters.

L111 Why did you run the stats on model predictions instead of on the raw data? I can’t easily find the goodness of fit information for this particular model, but even if it is quite good, model predictions are going to mask out real variability. If the model doesn’t explain the data well, then even more reason

not to use the predictions as a way to test statistical significance. Since you have the raw data, why not use it?

L119	Should be “to test for labile C limitation of microbial activity directly...” L136 Replace “simply” with “only”

L142 update to “a non-linear mixed effects model that explained the relationship between measured respiration and labile C addition and temperature....”

L183	Update to “...and uncertainty to our understanding of the relationships among...”

L210 This sentence is almost the same as L183. It is still not clear to me how you wish to link your findings of labile C limitation to shrub expansion, so this needs to be made more explicit.

L220 Should this be Boreal forest?

L260 combine sentences to read “watershed...using a CS215...”

L262 switch to past tense, as that is the tense used for other measurement descriptions

L266 add comma after 2018 and 2019

L297 delete “in”

L315 need comma after OH

L316 at what temperature were soils frozen/stored?

L317 this sentence is awkwardly worded. Change to “soils were thawed and rocks, large roots, and woody debris were removed before the soils were homogenized into a single composite with roughly three...”

L334 add total incubation time
L334 add comma after CO2

L379, L386, L401	change ~ to = ; ~ is an R code, not an equation.

L379 and L401 Each of the explanatory variables needs a regression coefficient. You have written the R code but not the equation. Should be something like:

Nutrient availability = B0 + B1*Temp + B2*Caddition + B3*Temp x Caddition + error

L386 This is how an interaction can be written in R, but it is not the correct mathematical expression. Should be something like:

resp =B0 + A*B^(T-10/10) + C*C addition + D*( A*B^(T-10/10) x C addition) + error

if I am understanding you correctly. It seems likely that the parameters A and B are not refit in this equation (and just the measured CO2 flux from L370 is used), but I don’t know for sure.

Reviewer #2 (Remarks to the Author):

After revision, this paper remains well written and an interesting analysis of the relationship between soil temperature, labile C availability, and overwinter microbial respiration.

The authors have thoughtfully addressed reviewers concerns. My primary concern with the original manuscript was lack of clarity in the methods section and contextualization of findings, both of which have been addressed or expanded in the revision.

I imagine Arctic and alpine researchers will find these results to be of high interest.

**Author rebuttal, second version:**

Reviewer #1 (Remarks to the Author):

Second review of Labile carbon limits late winter microbial activity near Arctic treeline by Sullivan and colleagues.

The authors have put a lot of work into thoroughly responding to reviewer comments, and the updated version is much improved as a result. However, there are still two main avenues for improvement.

The first is the statistical analyses. There are many claims in the text that are not backed up by statistical tests (or if they are, this is not made clear to the reader). As an example, L145 says “microbial respiratory responses to labile C addition [...] increased progressively with rising soil temperature (statistical evidence needed) [...]. Respiration, and the effect of C addition on respiration, declined during the three-month incubation (statistical evidence needed), especially at warmer temperatures (statistical evidence needed) [...].” I am not doubting that these trends exist, but I am arguing that they need to be substantiated. I have noted other questions about the statistical analyses in the line-by-line comments.

**All of the examples listed by Reviewer 1 pertain to our analysis of the microbial respiration data from the laboratory incubations. We had analyzed those data using a non-linear mixed effects (NLME) model. The reason why we chose this approach is because the respiratory response to rising temperature is exponential and would be poorly modeled if we assumed a linear relationship. The drawback to the NLME is that it is more difficult to examine the significance of individual coefficients in the model, which is essentially what Reviewer 1 requested. Thus, we have revised this analysis by log-transforming the respiration data to linearize the temperature-respiration relationship and then employed a linear mixed effects model, which is arguably a more robust statistical approach that lends itself to testing of the significance of individual model coefficients. We have updated the text to reflect this change in our analysis and we have included the results of the tests that Reviewer 1 requested.**

**This second look at the laboratory incubation respiration data inspired us to re-check all of our statistical tests. We made a couple minor revisions as part of that process, such has log-transforming the CO2 flux data from the field glucose additions and the NH4 data from the laboratory incubations. These changes didn’t have a qualitative effect on the results. They simply made our approach more defensible and our results more robust.**

**Finally, revisiting the respiration and nutrient availability data from the laboratory incubations revealed some potential differences in microbial responses to added C at below- versus above-freezing soil temperatures. As a result, we have decided to move the analyses that focus on just the below-freezing soil temperatures to the main text, while relegating the analyses that incorporate all soil temperatures to the Supplement. We think this is a sensible choice, because the focus of our manuscript is on overwinter microbial processes in frozen soils.**

The second is the Discussion. As written, it is pretty short, which I recognize is a feature of some Nature Communications papers. However, I think it could benefit from some discussion of the results of your nutrient analyses. As it is, a large chunk of that work is relegated to the results and supplementary material and the reader is left wondering about its broader significance. It is possible that these results could be woven into the existing discussion, or an additional paragraph could replace one of the existing.

**We have added a new paragraph to the Discussion that focuses on our soil nutrient analyses and their implications for competitive interactions among plants and between plants and microbes.**

Line-by-line comments:

L40-42 Is this sentence missing a word or punctuation? Perhaps it should read “...liquid water and solutes, which depress the freezing point and minimize the important physical constraints...”

**This sentence was not missing a word or punctuation, but we have replaced “as” with “because” to improve clarity.**

L52 Update to “greatest increases in temperature have been...”

**This change has been made.**

L56 change “show dramatic” to “indicate” because the regressions are only significant in one month, hardly dramatic.

**We have replaced “dramatic” with “strong”. This isn’t a reference to the number of months with significant trends, but to the significant February warming trend of +8 deg C/decade. We anticipate that the February warming trend will moderate as the length of our record grows. For that reason, we haven’t reported the slope and we haven’t drawn too much attention to the trend, but we think it is fair to say that it is indicative of strong winter warming.**

L57 change to “The combination of increasing winter temperatures and precipitation will lead to...”

**We have made this change, although we have used, “The combination of strong winter warming and increasing winter precipitation will lead to…”**

L59 do you mean in terms of the carbon cycle? Or what kinds of biological responses? Either way, should include some references here.

**This is a hypothesis and it is admittedly vague, but it foreshadows the following text of the discussion:**

**“Labile C limitation of microbial respiration may become more common if asymmetric warming leads to an imbalance whereby overwinter increases in microbial activity are not balanced by increases in microbial substrate use efficiency^22^ and/or vegetation productivity and associated labile C production. However, if development of labile C limitation leads to changes in nutrient cycling that yield greater N availability to plants, it is possible that increased vegetation productivity could lead to greater labile C inputs that might prevent further development of labile C limitation.”**

L77 remove “it was shown that” and update to read “...birch trees stimulated microbial

respiration...”

**We have reorganized this sentence, but would like to retain “were shown to”, because soil organic matter priming is an active area of research in the Arctic, with some studies highlighting its prevalence (e.g., Hartley et al. 2012), while others have found limited evidence of its importance (e.g., Lynch et al. 2018). Retaining “were shown to” helps to make it clear that these are the results of one study, rather than a phenomenon that has been observed throughout the tundra biome.**

L82 remove “it was shown that”

**We have made this change.**

L105 In L119 and L137, you give a brief overview of what you did before delving into the results.

Something similar would be helpful here; before this first sentence, you don’t even mention that measurements were taken for 3 winters.

**We have added a sentence describing what we did to the beginning of this paragraph.**

L111 Why did you run the stats on model predictions instead of on the raw data? I can’t easily find the goodness of fit information for this particular model, but even if it is quite good, model predictions are going to mask out real variability. If the model doesn’t explain the data well, then even more reason not to use the predictions as a way to test statistical significance. Since you have the raw data, why not use it?

**The question that we were asking here is whether CO2 flux at a given soil temperature varied across years, when measurements were made in late March. Soil temperature was not a controlled factor. It varied over space and time. We chose to use a soil temperature of -3 deg C because it was as close as possible to a common soil temperature across the three winters. Nevertheless, measurements of CO2 efflux that happened to correspond with a soil temperature of exactly -3 deg C were relatively rare. For this reason, it was not possible to subset the data to include only measurements made at exactly -3 deg C and conduct a simple statistical test using the raw data. Instead, we used a Monte Carlo approach to propagate error in the model fits and estimate the confidence intervals around the model predictions. Wider confidence intervals on the predictions are reflective of greater scatter in the raw data and a weaker fit of the model to the data. We are confident that this is an appropriate statistical approach to our question.**

L119 Should be “to test for labile C limitation of microbial activity directly...”

**We have made this change.**

L136 Replace “simply” with “only”

**We have made this change.**

L142 update to “a non-linear mixed effects model that explained the relationship between measured respiration and labile C addition and temperature....”

**This sentence has been changed in a manner consistent with the reviewer’s suggestion.**

L183 Update to “...and uncertainty to our understanding of the relationships among...”

**Good suggestion. We have made this change.**

L210 This sentence is almost the same as L183. It is still not clear to me how you wish to link your findings of labile C limitation to shrub expansion, so this needs to be made more explicit.

**We have re-written this sentence as follows, to make the link to shrub expansion and treeline advance more explicit:**

**“Our finding that labile C limitation of microbial respiration can develop over time in lightly frozen Arctic soils adds complexity and uncertainty to the positive feedbacks that are thought to reinforce shrub expansion and treeline advance into Arctic tundra.”**

L220 Should this be Boreal forest?

**We had left it as just “Boreal” with the aim of not excluding non-forested wetlands. We have replaced “forest” with “vegetation” in the subsequent sentence to be consistent with that goal.**

L260 combine sentences to read “watershed...using a CS215...”

**We have made this change.**

L262 switch to past tense, as that is the tense used for other measurement descriptions

**We have used present tense for all measurements that are still ongoing, including these long-term air temperature measurements.**

L266 add comma after 2018 and 2019

**We have made this change.**

L297 delete “in”

**We have made this change.**

L315 need comma after OH

**We have made this change.**

L316 at what temperature were soils frozen/stored?

**We have added this information (-20 deg C).**

L317 this sentence is awkwardly worded. Change to “soils were thawed and rocks, large roots, and woody debris were removed before the soils were homogenized into a single composite with roughly three...”

**We have made this change.**

L334 add total incubation time

**We have added this information.**

L334 add comma after CO2

**We have made this change.**

L379, L386, L401 change ~ to = ; ~ is an R code, not an equation.

**We have made this change in all instances.**

L379 and L401 Each of the explanatory variables needs a regression coefficient. You have written the R code but not the equation. Should be something like:

Nutrient availability = B0 + B1*Temp + B2*Caddition + B3*Temp x Caddition + error

**We have added the regression coefficients as requested.**

L386 This is how an interaction can be written in R, but it is not the correct mathematical expression.

Should be something like:

resp =B0 + A*B^(T-10/10) + C*C addition + D*( A*B^(T-10/10) x C addition) + error

**We have added the coefficients and the error term to this model specification as well.**

if I am understanding you correctly. It seems likely that the parameters A and B are not refit in this equation (and just the measured CO2 flux from L370 is used), but I don’t know for sure.

**This comment no longer applies, as we have revised this analysis to remove the Q10 function.**

Reviewer #2 (Remarks to the Author):

After revision, this paper remains well written and an interesting analysis of the relationship between soil temperature, labile C availability, and overwinter microbial respiration.

The authors have thoughtfully addressed reviewers concerns. My primary concern with the original manuscript was lack of clarity in the methods section and contextualization of findings, both of which have been addressed or expanded in the revision.

I imagine Arctic and alpine researchers will find these results to be of high interest.

**Reviewer comments, third version:**

Reviewer #1 (Remarks to the Author):

With this revision of the manuscript "Labile carbon limits late winter microbial activity near Arctic treeline," the authors have addressed all of my previous concerns. Overall I think it is an good manuscript and important contribution to our understanding of wintertime Arctic C cycling.

**Author rebuttal, third version:**
